# Angularly fused diaza-dinaphthopyrenes: regio-selective synthesis, crystal structures and isomer-dependent mechanochromic fluorescent properties†

**DOI:** 10.1039/d2sc05608a

**Published:** 2022-12-03

**Authors:** Yan Kou, Guangwu Li, Yi Han, Mengwei Li, Tingting Wang, Zhiyu Qu, Yulan Chen

**Affiliations:** a Department of Chemistry, Tianjin University Tianjin 300354 P. R. China yulanchen@jlu.edu.cn; b State Key Laboratory of Supramolecular Structure and Materials, College of Chemistry, Jilin University Changchun 130012 P. R. China; c Center of Single-Molecule Sciences, Institute of Modern Optics, Frontiers Science Center for New Organic Matter, College of Electronic Information and Optical Engineering, Nankai University Jinnan District Tianjin 300350 P. R. China; d College of Chemistry and Molecular Engineering, Peking University Beijing 100871 P. R. China

## Abstract

We report a one-pot synthesis of a series of unprecedented angular-fused diaza-dinaphthopyrene isomers (1,8-DNPy and 1,6-DNPy) in high yields, which are enabled by regio-selective Bischler–Napieralski cyclization to fuse two quinolone rings either on the same or opposite faces of a pyrene core. Benefiting from the high reactivity of the 1- and 8-positions of the pyrene ring, steric effect from substitution and remarkably different dipole moments, high ring closure selectivity for the 1,8-form *vs.* the 1,6-form up to 6 : 1 is achieved with ease of separation. With differentiated molecular symmetry, conformation, intermolecular interactions and aromaticity, the two kinds of regio-isomers exhibit distinct single-crystal structures and optoelectronic properties. Impressively, isomer-dependent mechanochromic fluorescent properties of these 2D-azaacenes are identified, which are unique in their turn-on fluorescence feature and contrasting spectral shifts. These findings allow facile and modular access to regio-specific 2D-N-heteroarenes, which provide a way to create innovative optical sensors with improved sensitivity and fruitful fluorescent properties.

## Abstract 2

A one-pot synthesis of the first angularly fused diaza-dinaphthopyrene isomers with central and axial symmetry is reported. They showed isomer-specific crystal structures, tunable optical and redox properties, and turn-on mechanochromic fluorescence.

## Graphical abstract

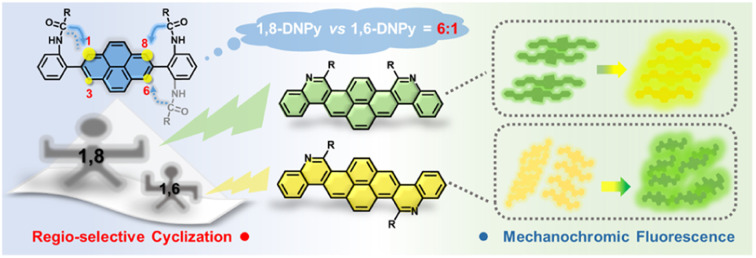

## Introduction

Acenes represent a family of polycyclic aromatic hydrocarbons (PAHs) with laterally fused benzene rings.^1^ They have become the functional material of choice for various applications such as organic electronics, biomedical imaging, sensors *etc.*^2^ In these scenarios, controlling the diverse field of acenes is of fundamental importance, whose key features lie in size, dimensionality and heteroaromatic doping.^3^ These factors have significant impacts on their electronic band structure, and optical and mechanical properties, and also influence their packing in the solid state.^3*f*^ In contrast to the development of long linear acenes,^1^ the strategy of precisely doping N atoms into large angular acenes could increase the electron affinity, dipole moment and intermolecular interactions, and more importantly, stabilize the frontier molecular orbitals of the resultant π-extended 2D-azaacenes.^4^ However, N-doping and π-extension both raise a considerable issue regarding regio-selective synthesis.

**Fig. 1:**
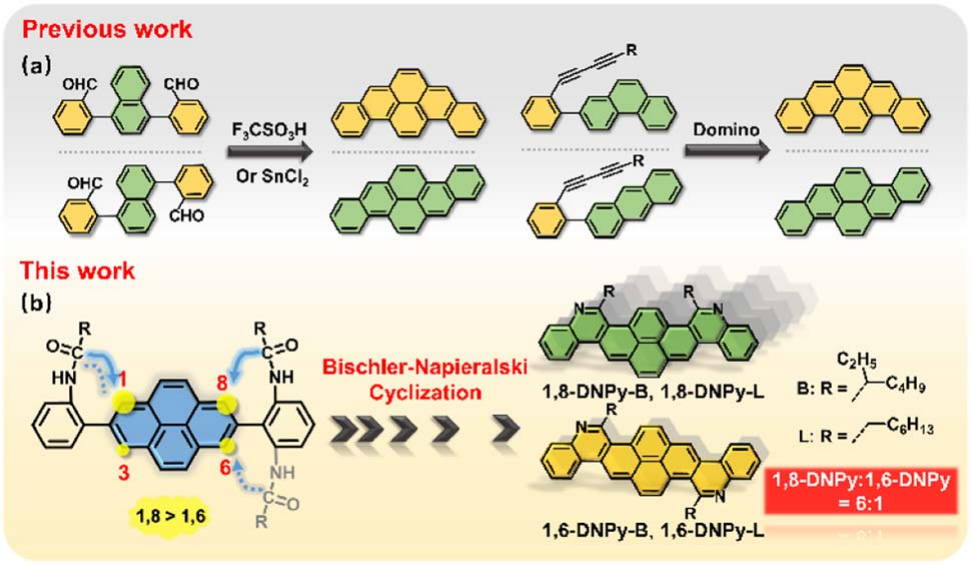
(a) Examples of reported strategies towards dibenzopyrene isomers. (b) Schematic illustration of the one-pot, regio-selective synthesis of diaza-dinaphthopyrene isomers in this work.

Pyrene is a versatile fluorophore to build up larger acenes.^5^ Great research endeavors have been devoted to ring closure reactions toward pyrene-cored acenes.^6^ A notable example is dibenzopyrenes (Fig. 1a). Their isomers can be prepared by a dehydrative π-extension cyclization of the corresponding aromatic aldehydes,^7*a*,*b*^ or domino reactions of buta-1,3-diynes.^7*c*^ Such isomerization relies on the respective reactions from different precursors. So far, one-pot synthesis of regio-selective angular azaacenes from pyrene derivatives and illustration of their isomer-dependent properties are limited, mostly due to the lack of sophisticated synthetic protocols. Note that the pyrene ring presents different reactivities in the same molecule,^5,8^ which raises synthetic difficulties in accessing some positions, but in turn, offers opportunities for regio-specific electrophilic substitution or N-doping from a single precursor.

Our previous work has demonstrated that Bischler–Napieralski cyclization is a powerful approach to N-doping of PAHs, offering aza-PAHs such as phenanthridine, thiophene fused 1,10-phenanthroline, diazapyrene, 5,6,12,13-tetraazaperopyrenes, *etc.*^9^ This reaction includes acylation and intramolecular electrophilic substitution. As for the pyrene ring, it is well known that electrophilic substitution occurs preferentially at its 1,3,6,8-positions. When one of the four sites is occupied, the others will react in the order of 8- > 6- > 3- positions.^5,10^ In this regard, we expect that Bischler–Napieralski cyclization of pyrene-involved bis-amide precursors will have high selectivity at its 1- and 8- positions, so that the unprecedented, regio-selective synthesis of diaza-dinaphthopyrenes is very promising (Fig. 1b).

Herein, we describe one-pot access to regio-isomeric diaza-dinaphthopyrenes with central and axial symmetry respectively, by implementing regio-selective Bischler–Napieralski cyclization reaction as a key step (1,8-DNPy-B, 1,6-DNPy-B, 1,8-DNPy-L, and 1,6-DNPy-L, Fig. 1b). Although the four fluorophores possess analogous conjugated backbones, our study uncovered that their different substitutions, N-doping and annulation manners played critical roles in controlling regio-selectivity, intermolecular interactions, and electronic and optical properties. Disparately different crystal structures, and photophysical and mechano-responsive properties of these isomers were estimated. The 1,6-species display larger dipole moments, narrower band gaps, higher fluorescence yields and more compact stacking than the 1,8-isomers. Impressively, the highest ring closure selectivity was observed for branched chain substituted 1,8-DNPy-B, which exhibit distinct mechanochromic fluorescent (MCF) properties with unusual enhanced fluorescence quantum efficiency after mechanical disturbance. In contrast, similar turn-on MCF behaviours yet with a rare hypsochromic shift were detected for the 1,6-counterpart.

## Results and discussion

**Fig. 2:**
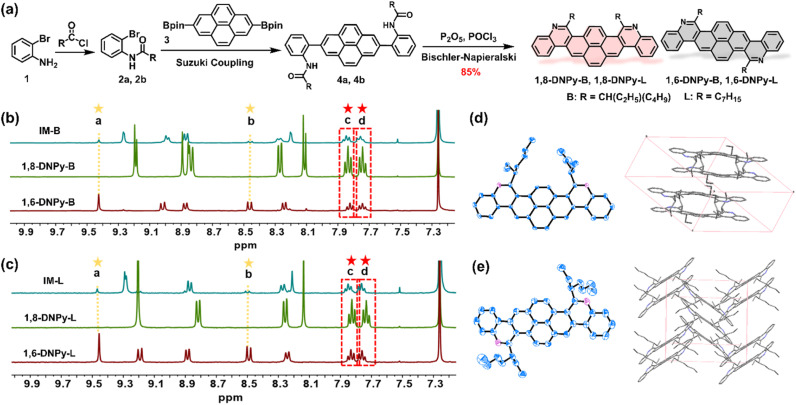
Synthesis and characterization of 1,8-DNPys and 1,6-DNPys. (a) The synthetic route of the four diaza-dinaphthopyrenes. Sections of ^1^H NMR spectra of (b) the as-prepared mixture of 1,8-DNPy-B and 1,6-DNPy-B, and 1,8-DNPy-B and 1,6-DNPy-B; (c) the as-prepared mixture of 1,8-DNPy-L and 1,6-DNPy-L, 1,8-DNPy-L and 1,6-DNPy-L. ORTEP-3 diagrams for the single-crystal structures and packing modes of (d) 1,8-DNPy-B and (e) 1,6-DNPy-B. Thermal ellipsoids are drawn at the 50% probability level. Hydrogen atoms have been omitted for clarity.

The structures and synthetic routes of the target diaza dinaphthopyrenes are outlined in Fig. 2a. They are isomeric pyrene-cored acenes with two nitrogen atoms regio-selectively doped at different sites, attached with branched or linear alkyl chains. The key bis-amide precursors 4a and 4b were prepared by Suzuki cross-coupling of 2 with 2,7-bis-(Bpin)pyrene (3). Subsequently, two-fold Bischler–Napieralski cyclization of 4 on the pyrene unit in the presence of refluxed P_2_O_5_/POCl_3_ led to a set of mixtures of 1,8-DNPy and 1,6-DNPy with the total yield of isomers up to 85%. Due to the freely rotated C–C bonds in 4a and 4b, Bischler–Napieralski cyclization likely proceeded on the same side of their long axis to generate the axial symmetric 1,8-DNPy-B and 1,8-DNPy-L, otherwise, when close on the opposite side, centrosymmetric 1,6-DNPy-B and 1,6-DNPy-L could be formed.

NMR analysis of the as-prepared mixtures revealed that 1,8-DNPys were preferentially produced and the selectivity was additionally dependent on the peripheral chains. Particularly, 1,8-DNPy-B with branched chains was yielded with the highest ring-closure regio-selectivity. Each separated isomer showed only one set of resonances, with characteristic peaks either split into two parts or shifted to a high field relative to the signals from their mixture samples. This finding indicated the existence of significant interactions between 1,8-DNPys and 1,6-DNPys (Fig. S1†). According to the integrated areas of characteristic proton peaks assignable to 1,6-DNPy-B (labeled as a and b in Fig. 2b) and to the as-prepared mixture (labeled as c and d), the molar ratio of 1,8-DNPy-B/1,6-DNPy-B in the as-prepared mixture was estimated to be 6 : 1. Similarly, the ratio of 1,8-DNPy-L/1,6-DNPy-L is about 4 : 1 (Fig. 2c). As control experiments, Bischler–Napieralski cyclizations carried out in non-polar solvents (*e.g.*, toluene and CCl_4_) instead of POCl_3_, or with other branched chain attached amide precursor displayed similar regio-selectivity of 1,8- and 1,6-forms (Fig. S2 and S3†). Additionally, all cyclizations were done in fully dissolved states. Thus, the factors of polarity and solubility to favor the generation of 1,8-forms could be excluded. As a result, the dominant formation of 1,8-DNPy-B can be mainly ascribed to the high reactivity of the 1- and 8-positions of the pyrene ring. Synergistically, the steric hindrance from branched chains differentially influenced the bifurcated Bischler–Napieralski cyclization of 4a, thus reinforcing the regio-selective preparation of 1,8-DNPy-B.

Besides the high regio-selectivity, 1,8-DNPys and 1,6-DNPys have remarkably different dipole moments and polarity owing to their contrasting symmetries. For instance, 1,8-DNPy-L is an axisymmetric molecule, showing a large dipole moment (2.49 Debye) along the short molecular axis. In contrast, 1,6-DNPy-L is centrosymmetric with almost overlapped negative and positive charge centers.^13^ Its dipole moment was calculated to be as low as 0.64 Debye (Table S5†). The two factors facilitated the separation of these isomers by quick silica gel column chromatography (Experimental details in the ESI†). All compounds were unambiguously characterized by ^1^H NMR and ^13^C NMR spectroscopy as well as mass spectrometry. And their good thermal stability was confirmed by thermogravimetric analysis (Fig. S4†). The 5% weight loss temperature of each nitrogen-doped molecules is close to 400 °C, which is higher than that of the non-doped acene analogues.^10*c*^ Interestingly, the differential scanning calorimetry (DSC) and polarized optical microscopic (POM) measurements showed a mesophase transition in both 1,8-DNPy-L and 1,6-DNPy-L (Fig. S5 and S6†). For example, typical focal conic texture was detected in 1,8-DNPy-L.

Single crystals of 1,6-DNPy-B and 1,8-DNPy-B suitable for X-ray crystallography were grown by slow evaporation of chloroform/isopropyl alcohol. Both molecules display slightly distorted π-skeletons with torsion angles in the range of 10–15° in their crystal structures (Fig. S7 and S8†). Disparately different packing modes were observed for the two regio-isomers. The 1,8-DNPy-B crystal belongs to the triclinic space group *P*1̄, with a dimer feature by an antiparallel and dislocated arrangement of 1,8-DNPy-B (Fig. 2d, Table S1†). The packing is driven by multiple intermolecular interactions, such as π⋯π, C–H⋯C(CH_3_)–H and C–H⋯π interactions, with distances in the range of 2.398–3.478 Å. In contrast, as the molecules slipped along the diagonal direction, 1,6-DNPy-B adopts an edge-to-face herringbone-packing pattern in the monoclinic space group *P*2_1_/*n* (Table S2†). In each cell unit, the adjacent two and three molecules stack in parallel with the dihedral angle between the molecular planes of ∼67° (Fig. 2e). Compared to 1,8-DNPy-B that is packed by π⋯π, C–H⋯C(CH_3_)–H and C–H⋯π interactions with distances in the range of 2.398–3.478 Å, the 1,6-DNPy-B crystal exhibits more π⋯π, C(CH_3_)–H⋯C(CH_3_)–H, C(CH_2_)–, H⋯C(CH_3_)–H, C⋯C and C–H⋯π interactions with closer contact in the range of 2.373–3.388 Å extending in two directions, manifesting a more compact packing in 1,6-DNPy-B.^11^

**Fig. 3:**
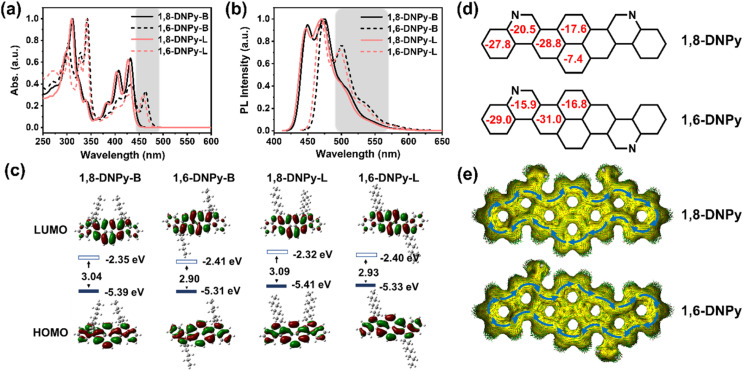
(a) Normalized UV-vis absorption spectra of the four 1,8-DNPys and 1,6-DNPys in THF (1 × 10^−5^ M). (b) Fluorescence spectra of 1,8-DNPy-B (*λ*_ex_ = 410 nm), 1,6-DNPy-B (*λ*_ex_ = 429 nm), 1,8-DNPy-L (*λ*_ex_ = 410 nm) and 1,6-DNPy-L (*λ*_ex_ = 429 nm) in THF (1 × 10^−5^ M). (c) Calculated molecular orbitals of the four 1,8-DNPys and 1,6-DNPys (TD-DFT, B3LYP/6-31G*). (d) NICS(1)_*zz*_ values (red) and (e) ACID plots of 1,8- and 1,6-diaza-dinaphthopyrene isomeric backbones. Blue arrows indicate the peripheral ring currents (B3LYP/6-31G(d,p)).

In dilute THF solution, the four 2D-azaacenes show well-resolved absorption bands ranging from 250 to 500 nm, which represent a sign of rigidified π-extended pyrene skeletons and are attributed to intramolecular π–π* and n–π* transitions (Fig. 3a). The longest-wavelength absorption maximum of 1,8-DNPy-B, 1,6-DNPy-B, 1,8-DNPy-L and 1,6-DNPy-L is at 432, 463, 427, 458 nm, respectively. All the molecules emitted blue-green fluorescence with their FL spectra featuring small Stokes shifts (13–20 nm) and mirror images of their absorption bands (Fig. 3b), reflecting the high rigidity of the backbone. Remarkably, both 1,6-DNPy-B and 1,6-DNPy-L solutions display bathochromic shifts of their absorption and emission bands of ∼35 nm and much higher fluorescence quantum yields (25.31% and 30.70%, relative to coumarin 6),^12^ compared to their 1,8-counterparts (9.56% and 11.88%, Table 1). All the different photophysical properties are understandable since compared to 1,8-DNPys, the central part of 1,6-DNPys can be regarded as a 2D-angular bis-benzo[*g*]isoquinoline to offer a higher conjugation degree for 1,6-DNPys. This is also consistent with the observation of strong π–π interactions existing in 1,6-DNPy-B crystals. In addition, all these spectra are independent of solvents (Fig. S9†), suggesting the absence of intramolecular charge transfer transition in these molecules. On the other hand, N-atoms doped in acenes are easily protonated,^9*b*^ endowing these azaacenes with acid-responsive feature, with appreciably changed optical properties (Fig. S10†).

**Table 1:** Optical and electronic properties of 1,8-DNPy-B, 1,6-DNPy-B, 1,8-DNPy-L and 1,6-DNPy-L

	UV-vis absorption		Fluorescence	*Φ* b(%)/*Φ*^c^(%)	*E*_g_ d (eV)	HOMO^e^ (eV)	LUMO^e^ (eV)
	*λ*_abs_ a (nm)	*ε*	*λ*_em_ a (nm)				
1,8-DNPy-B	432	5.4 × 10^4^	450, 475, 510	9.56/34.04	2.71	−5.58	−2.87
1,6-DNPy-B	463	4.0 × 10^4^	476, 505, 536	25.31/22.25	2.55	−5.49	−2.94
1,8-DNPy-L	427	8.4 × 10^4^	447, 472, 506	11.88/20.44	2.74	−5.64	−2.90
1,6-DNPy-L	458	3.5 × 10^4^	472, 503, 535	30.70/15.96	2.58	−5.53	−2.95

a The longest absorption and emission wavelength in dilute THF solution ([*c*] = 10^−5^ M). *ε* represents the molar extinction coefficient in M^−1^ cm^−1^.

b The relative fluorescence quantum yield (*Φ*_F_) was measured using coumarin 6 in CH_2_Cl_2_ as a standard (*Φ*_F_ = 76%).

c Absolute fluorescence quantum efficiency (*Φ*_F_), determined using a calibrated integrating sphere for solids.

d Estimated from the onset of the UV-vis absorption spectrum.

e *E*_HOMO_ = −(4.74 + *E*_ox_), *E*_LUMO_ = *E*_HOMO_ + *E*_g_, *E*_ox_ is the onset potential of the first oxidation wave *vs. E*_Fc/Fc^+^_.

Next, the redox properties of the four compounds were examined by cyclic voltammetry in CH_2_Cl_2_ (Fig. S11,†Table 1). Within the solvent window, 1,8- and 1,6- isomers exhibit irreversible oxidation peaks. The oxidation potential of 1,8-DNPy-B (0.84 eV) is about 0.09 eV higher than that of 1,6-DNPy-B (0.75 eV). Their HOMO levels were calculated to be −5.58 eV and −5.49 eV, respectively. Meanwhile, 1,8-DNPy-B and 1,6-DNPy-B both exhibit quasi-reversible reduction peaks, indicating their electron-deficient properties.^9*a*^ The LUMO levels of 1,8-DNPy-B and 1,6-DNPy-B were then estimated to be −2.87 and −2.94 eV, respectively. 1,8-DNPy-L and 1,6-DNPy-L exhibit a qualitatively similar isomer-dependent optoelectronic trend. Thus, the reduced band gaps of 1,6-DNPys as compared to 1,8-DNPys were estimated, due to their lower LUMO and higher HOMO levels. All the photophysical and redox data are summarized in Table 1.

To gain insights into the electronic structures and aromaticity of these isomers, time-dependent density functional theory (TD-DFT) calculations at the B3LYP levels of theory under vacuum conditions were performed. The calculated UV-vis absorption profiles (B3LYP/6-31G*) of 1,8-DNPy and 1,6-DNPy displayed resemble vibration bands and profound red-shifted absorption (Δ*λ* ∼ 30 nm) from 1,8-DNPy to 1,6-DNPy, which were in line with the experimental spectra (Fig. 2a, S12 and S13†). The longest absorption bands are assigned to the HOMO–LUMO transition (Tables S3 and S4†). For all isomers, electrons in the HOMO delocalize over the whole conjugated skeleton, whereas those of the LUMO are mostly positioned on the pyrene core, corresponding to the electron-deficiency of quinolone rings (Fig. 3c). Additionally, similar to the experimental results, DFT calculations supported the concurrent increase in the HOMO level and decrease in the LUMO level, which led to a reduction in band gap by 0.14 eV going from 1,8-DNPys to 1,6-DNPys. Besides, nucleus-independent chemical shift (NICS) and anisotropy of the induced current density (ACID) analysis (B3LYP/6-31G(d,p)) results are presented in Fig. 3d and e. All fused rings in 1,8-DNPy and 1,6-DNPy show negative NICS(1)_*zz*_ values. The magnitudes of NICS(1)_*zz*_ values are more negative in 1,6-DNPy than in 1,8-DNPy, suggesting stronger diatropicity of 1,6-DNPy.^1^ This observation corroborated the experimental finding that 1,6-DNPys possessed a higher conjugation degree. Both 1,8-DNPy and 1,6-DNPy exhibit interesting global aromaticity with clockwise ring current along the periphery of the whole backbone according to their ACID plots. The π systems were thus efficiently enlarged by our methods.

**Fig. 4:**
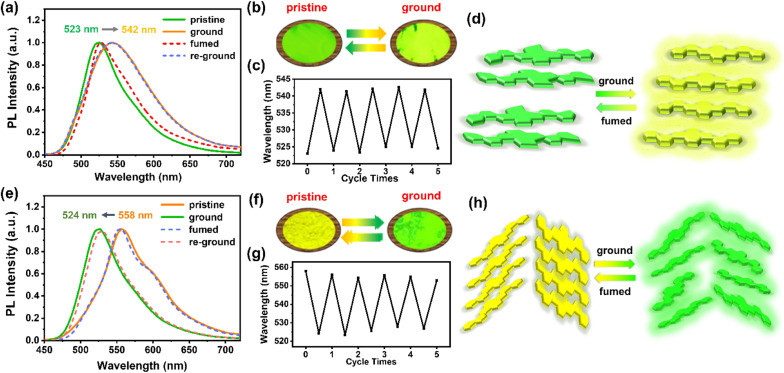
MCF properties of (a–d) 1,8-DNPy-B and (e–h) 1,6-DNPy-B. Fluorescence emission spectra of (a) 1,8-DNPy-B (*λ*_ex_ = 410 nm) and (e) 1,6-DNPy-B (*λ*_ex_ = 429 nm) in different solid states. The photographs of (b) 1,8-DNPy-B and (f) 1,6-DNPy-B in different solid states under UV illumination of 365 nm. Switching of the maximum emission wavelength of (c) 1,8-DNPy-B (523 to 542 nm) and (g) 1,6-DNPy-B (558 to 524 nm) by repeated grinding and fuming processes. The speculative mechanisms of (d) 1,8-DNy-B and (h) 1,6-DNPy-B.

Intriguingly, although the highly rigid and conjugated 2D-azaacene core is undesirable for mechano-responsiveness of the resultant materials, both 1,8-DNPy-B and 1,6-DNPy-B exhibited distinct MCF properties with an extraordinary turn-on feature. As shown in Fig. 4, the pristine crystalline samples of 1,8-DNPy-B and 1,6-DNPy-B were emissive with bright green or yellow fluorescence. After grinding, the emission band of 1,8-DNPy-B became broad and shifted bathochromically from the green to the yellow region (Fig. 4a and b). Notably, the absolute fluorescence quantum efficiency (*Φ*_F_) enhanced considerably from 34.04% to 44.75%. In contrast, the emission peak of the ground 1,6-DNPy-B sample displayed an opposite blue shift (from 558 nm to 524 nm, Δ*λ* = 34 nm) along with the disappearance of the fine structure and slight enhancement of *Φ*_F_ (from 22.25% to 24.30%, Fig. 4e and f). The fluorescence lifetime (*τ*) of both increased after grounding, *e.g.*, from 11.57 ns to 13.99 ns for 1,8-DNPy-B and from 2.96 ns to 7.65 ns for 1,6-DNPy-B (Fig. S18†). After fuming the ground powders with CH_2_Cl_2_ vapor, their original luminescence could be recovered. The fluorescence switching stimulated by mechanical force and CH_2_Cl_2_ vapor could be repeated many times without fatigue (Fig. 4c and g). All the fluorescence switches were sufficiently obvious to be easily distinguished by the naked eye. It is worth mentioning that organic MCF materials primarily experienced weakened fluorescence intensity and spectral redshifts after mechanical treatment.^14^ Herein, such a turn-on feature in terms of their pronounced enhancement of luminescence and elongated lifetime is unique, which is essentially important to offer innovative force sensors with improved sensitivity. This finding, together with the unusual hypsochromic shifted emission from 1,6-DNPy-B, extended our knowledge of the structures and functionalities of angularly fused azaacenes; particularly highlighting that diaza-dinaphthopyrene is a versatile fluorophore to produce isomer-specific, unconventional sensing signals.

The isomer-dependent MCF mechanism was further explored. First, as shown in Fig. S16,† UV-vis spectra of 1,8-DNPy-B and 1,6-DNPy-B before and after ground are almost unchanged, correspondence to the well-preserved chemical structures of these azaacenes. Second, DSC curves (Fig. S14b and S15b†) of the ground 1,8-DNPy-B and 1,6-DNPy-B displayed a cold–crystallization transition peak at 69.5 °C and 77.5 °C, respectively, suggesting that the ground powders were in a thermodynamically metastable state.^9*c*,13*c*^ Besides, powder X-ray diffraction (PXRD) profiles (Fig. S14a and S15a†) manifested the reversible phase transitions between the ordered crystalline state and amorphous one for 1,6-DNPy-B.^9*c*^ But under the same conditions, changes in diffraction profiles for 1,8-DNPy-B were not that significant. Therefore, mechanically triggered amorphous state conversion of 1,8-DNPy-B was not the dominant factor for its turn-on and bathochromic shifted emission. Otherwise, when referring to the single-crystal structure, we could see that 1,8-DNPy-B represented a more twisted conformation and stacked antiparallel in a dimer form, which might readily be disturbed into a planar form.

Collectively, by considering the different molecular conformations, intermolecular packing modes and phase transitions of the two isomers,^15^ we assumed that the mechanical force-induced planarization of 1,8-DNPy-B would be responsible for the red-shifted and turn-on emission (Fig. 4d). Nevertheless, as for the herringbone stacked 1,6-DNPy-B, mechanical disturbance could easily slip the adjacent molecules, reduce the degree of their π-overlapping, and lead to a less-ordered state and weaker π–π interactions. This proposed mechanism helps us to explain its unusual blue-shifted and enhanced fluorescence upon grinding (Fig. 4h). It should be mentioned that the linear chain substituted analogs 1,8-DNPy-L and 1,8-DNPy-L were not mechano-responsive, due to their strong tendency of aggregation to hamper the mechanical disturbance^16^ (Fig. S17†). Overall, we can deduce that the regio-selective N-doping and substitution with bulky side groups appear as efficient design principles to endow diaza-dinaphthopyrenes with sensitive and tunable MCF properties.

## Conclusions

We have developed a straightforward method to synthesize isomeric diaza-dinaphthopyrenes *via* selective Bischler–Napieralski cyclization. This strategy is unique in terms of the ease of preparation (one pot, bifurcated synthesis of two regio-isomers in high yields), good regio-selectivity (ratio of 1,8-DNPy/1,6-DNPy up to 6 : 1), and relatively simple purification (ease of column chromatography due to the distinct polarity of the two isomers). As deduced from ^1^H NMR, single-crystal structures, optical and redox properties and DFT calculation results, the N-doping and aromatic fusion manners, together with the steric substitutions, offered a practical molecular design strategy to tune the regio-selectivity, intermolecular interactions, electron structures and aromaticity. It is worth mentioning that although these fluorophores possessed rigidified eight fused rings, 1,8-DNPy-B and 1,6-DNPy-B can sensitively respond to mechanical grinding with distinctly opposite MCF properties, displaying either bathochromic- or hypochromic-shifted emission from the ground samples. Importantly, both of them exhibit mechanically induced enhanced fluorescence. This is a fascinating merit overstepping most reported cases, enabling a highly sensitive reporting of mechanical disturbance. We expect that the current design and synthetic strategy can be applied to various other angularly fused N-heteroarenes with tunable optoelectronic properties and extraordinarily sensitive MCF signals.

## Data availability

All the data supporting this article have been included in the main text and the ESI.†

## Author contributions

The manuscript was written with the contributions of all authors. All authors have approved the final version of the manuscript.

## Conflicts of interest

There are no conflicts to declare.

## Supplementary Material

SC-014-D2SC05608A-s001

SC-014-D2SC05608A-s002
