# Supplementary material for: Angularly fused diaza-dinaphthopyrenes: regio-selective synthesis, crystal structures and isomer-dependent mechanochromic fluorescent properties
Source: Chem Sci. 2022 Dec 3;14(3):668–74. doi: 10.1039/d2sc05608a (PMC9848161; doi:10.1039/d2sc05608a)
Supplement: SC-014-D2SC05608A-s001 [file SC-014-D2SC05608A-s001.pdf]

Electronic Supplementary Material (ESI) for Chemical Science.
This journal is © The Royal Society of Chemistry 2022

Supporting Information

Angularly Fused Diaza-Dinaphthopyrenes: Regio-Selective Synthesis, Crystal Structures and Isomer-Dependent Mechanochromic Fluorescent Properties

Yan Kou,[a] Guangwu Li,[c] Yi Han,[a] Mengwei Li,[a] Tingting Wang,[a] Zhiyu Qu[d] and Yulan Chen*[a,b]

a. Department of Chemistry, Tianjin University, Tianjin 300354 (P. R. China). E-mail: yulanchen@jlu.edu.cn

b. State Key Laboratory of Supramolecular Structure and Materials, College of Chemistry, Jilin University, Changchun 130012 (P. R. China).

c. Center of Single-Molecule Sciences, Institute of Modern Optics, Frontiers Science Center for New Organic Matter, College of Electronic Information and Optical Engineering, Nankai University, Jinnan District, Tianjin 300350 (P. R. China).

d. College of Chemistry and Molecular Engineering, Peking University, Beijing 100871 (P. R. China).

Table of Contents

1. **Experimental Section** 3-7
2. **Results and Discussion**
   Self-assembled gels (Figure S1) 8
   Control Bischler-Napieralski cyclizations (Figure S2, S3) 8
   TGA curves (Figure S4) 9
   Characterization of phase transition (Figure S5, S6) 9-10
   Single crystal information (Figure S7, S8, Table S1, S2) 10-11
   Optical properties (Figure S9) 12
   Acid-responsive properties (Figure S10) 13
   Redox properties (Figure S11) 13
   TD-DFT calculations (Figure S12, S13, Table S3-S5) 14-15
   Mechanochromic fluorescent properties (Figure S14-S16) 16
   XRD curves (Figure S17) 17
   Fluorescence decay curves (Figure S18) 17
   NMR and MS spectra of all compounds (Figure S19-S40) 18-25
   Computational data for compounds 25-34
3. **References** 34

Experimental Section

Materials and methods

General. Unless otherwise noted, the commercially available reagents were purchased from commercial suppliers and used without further purification. Dichloromethane (CH_2Cl_2) was distilled over CaH_2. Tetrahydrofuran (THF) was purified by distillation from benzophenone and sodium. All reactions were monitored by TLC with silica gel 60 F254. Column chromatography was carried out on silica gel (200-300/100-200 mesh). The catalyst precursor $Pd(PPh_3)_4$ was prepared according to the literature,[1] and stored in a Schlenk tube under nitrogen atmosphere.

Measurements. ^1H NMR (400 MHz) and ^{13}C NMR (101 MHz) spectra were recorded in deuterated solvents on a 400 MHz Bruker AV400 spectrometer. The high resolution electrospray mass spectra (HR-ESI-MS) of products were recorded on a micrOTOF-Q II 10204 mass spectrometer. Matrix assisted laser desorption ionization time-of-flight mass spectroscopy (MALDI-TOF) was performed on a Bruker Autoflex speed TOF/TOF mass spectrometer using dithranol as the matrix. Melting points were measured with an SGW X-4A apparatus. The UV-vis absorption spectra were obtained on a PerkinElmer Lambda 750 UV/VIS/NIR spectrophotometer. Fluorescence spectra were recorded on a Hitachi F-7000 fluorescence spectrophotometer. Thermogravimetric analyses (TGA) were carried out using a TA Instruments Q-50 with a heating rate of 10 °C/min. Differential scanning calorimetry (DSC) measurements were carried out with a TA DSC Q-20 under a nitrogen atmosphere at a heating rate of 10 °C/min. Optical and polarized optical microscopic images were recorded with an Olympus BH-2 optical microscope with a Mettler hot stage (FP-52) and an automatic camera. The powder XRD patterns were obtained with a Rigaku SmartLab (9 kW) X-ray diffractometer. The single crystal X-ray diffraction was recorded on Rigaku XtaLAB FRX (2.97 kW) FR-X super bright X-ray with rotating anode assembly Cu in 160 K by ω scan mode, the effective focal size was approximately 70 microns. The absolute fluorescence quantum yields were measured by using an absolute PL quantum yield spectrometer (Edinburg FLS-980 fluorescence spectrometer) with a calibrated integrating sphere. The fluorescence lifetime measurements were recorded on FLS-1000 spectrometer (Edinburgh Instruments) using a laser with excitation wavelength of 375 nm and time-correlated single photon counting (TCSPC). Cyclic voltammetric experiments were carried out using a CHI 660E electrochemical workstation (CH Instruments, ChenHua, Shanghai, China). All voltammograms were acquired at room temperature. A standard three electrode electrochemical cell arrangement was employed using Pt carbon as working electrode, a Pt wire as counter electrode, and Ag/Ag^+ as reference electrode in 0.1 M CH_2Cl_2 containing tetrabutylammonium hexafluorophosphate ($TBAPF_6$) as the supporting electrolyte with a sweep rate of 100 mV s^{-1}. The potentials are reported vs the Fc^+/Fc redox couple as a standard. Density functional theory (DFT) calculations and time-dependent density functional theory (TD-DFT) calculations were performed in Gaussian 09 software at the B3LYP functional with the 6-31G* basis set level. Nucleus-independent chemical shifts (NICS) calculations of the compounds are at the B3LYP/6-31G(d,p) computational level and were all carried out using the Aroma package according to published procedures.[2,3] All the $NICS(1)_{zz}$ that are reported here are obtained from NICS-scan and the σ-only model.[4] The $NICS(1)_{zz}$ values are derived from the sigma only model. Anisotropy of the induced current density (ACID) plots (B3LYP/6-31G(d,p)) was calculated using

the method developed by Herges.[5] The result visualization was carried out by using POV-Ray v3.7 software.

Fluorescence emission spectrum and relative fluorescence quantum yield measurement: Using the Hitachi F-7000 fluorescence spectrophotometer, the test was carried out at room temperature, and the calibration curve was selected in the fluorescence spectrum test. The calculation formula of the relative fluorescence quantum yield was as follows:

$$\Phi = \Phi_r \frac{I}{I_r} \frac{A_r}{A} \frac{\mathrm{n}^2}{\mathrm{n_r}^2}$$

Among them, Φr and Φ are the fluorescence quantum yields of the standard product and the sample to be tested, I_r and I are the integrated area under the emission spectrum curve of the standard product and the sample to be tested respectively, and $\mathrm{n_r}$ and n are the standard product and the sample to be tested respectively. The refractive index of the solvent at room temperature, A_r and A are the absorbance at the excitation wavelength of the standard and the sample to be tested, respectively.

Experimental Details

General procedure for preparation of (2a). A mixture of 2-ethylhexanoyl chloride (2.0 g, 12.21 mmol) and CH_2Cl_2 (15 mL) was added dropwise to a mixture of **1** (2.0 g, 11.63 mmol), TEA (3.2 mL, 23.02 mmol) and CH_2Cl_2 (30 mL) at 0 °C in an ice water bath. After stirring at room temperature overnight, the reaction was quenched with water and extracted with CH_2Cl_2. The combined organic extracts were dried over anhydrous $MgSO_4$ and concentrated under reduced pressure. The residue was purified by chromatography on silica gel column eluting with PE/DCM (v/v, 4/1) to afford **2a** as a white powder (2.87 g, 83%).

^{1}H NMR ($CDCl_3$, 400 MHz, ppm): δ 8.37 (d, J = 8.4 Hz, 1H, ArH), 7.63 (s, 1H, NH), 7.53 (dd, J_{12} = J_{34} = 1.7 Hz, J_{23} = 7.4 Hz, 1H, ArH), 7.31 (m, J = 7.3 Hz, 1H, ArH), 6.97 (m, J = 7.0 Hz, 1H, ArH), 2.19 (m, 1H, CH), 1.72 (m, 2H, CH_2), 1.58 (m, 2H, CH_2), 1.34 (m, 4H, CH_2), 0.98 (t, 3H, CH_3), 0.89 (m, 3H, CH_3). ^{13}C NMR ($CDCl_3$, 101 MHz, ppm): δ 174.34, 135.69, 132.15, 128.36, 125.06, 122.13, 113.44, 51.03, 32.57, 29.77, 26.23, 22.76, 13.96, 12.09. MALDI-TOF-MS, m/z: calculated for $C_{14}H_{20}BrNO$ $[M+Na]^+$: 321.214; found, 321.128.

General procedure for preparation of (2b). 2b was synthesized following the procedure for **2a**, with decanoyl chloride (2.33 g, 12.21 mmol) in CH_2Cl_2 (10 mL), **1** (2.0 g, 11.63 mmol) and TEA (3.2 mL, 23.02 mmol) in CH_2Cl_2 (30 mL) as reagents. After the reaction was completed, the post-treatment method was as same as above, the residue was purified by chromatography on silica gel column eluting with PE/DCM (v/v, 6/1) to afford **2b** as a white powder (2.77 g, 80%).

^{1}H NMR ($CDCl_3$, 400 MHz, ppm): δ 8.34 (d, J = 8.3 Hz, 1H, ArH), 7.64 (s, 1H, NH), 7.51 (dd, J_{12} = J_{34} = 1.7 Hz, J_{23} = 7.5 Hz, 1H, ArH), 7.29 (m, J = 7.3 Hz, 1H, ArH), 6.95 (m, J = 7.0 Hz, 1H, ArH), 2.42 (t, 2H, CH), 1.74 (m, 2H, CH_2), 1.39-1.33 (m, 8H, CH_2), 0.88 (t, 3H, CH_3). ^{13}C NMR ($CDCl_3$, 101 MHz, ppm): δ 171.41, 135.76, 132.18, 128.38, 125.04, 121.98, 113.30, 38.03, 31.68, 29.16, 29.03, 25.57, 22.62, 14.09. MALDI-TOF-MS, m/z: calculated for $C_{14}H_{20}BrNO$ $[M+Na]^+$: 321.214; found, 321.133.

General procedure for preparation of (3).[6] In a nitrogen-filled glove box, [{Ir (μ-OMe)cod}$_2$] (60 mg, 0.09 mmol), 4,4'-di-tert-butyl-2,2'-bipyridine (dtbpy, 48 mg, 0.18 mmol), and B_2pin_2 (100 mg, 0.39 mmol) were dissolved in THF (5 mL). The mixture was added to a Young's tube containing pyrene (1.80 g, 8.90 mmol) and B_2pin_2 (4.86 g, 19.1 mmol). After addition of THF (10 mL), the tube was sealed and the reaction mixture was stirred at 80 °C for 16 h. Then, the reaction mixture was passed through a 5 cm silica plug (eluent: CH_2Cl_2) and the solvent was removed under reduced pressure. The pale-yellow residue was purified by refluxing hexane (100 mL) to afford **3** (3.63 g, 90%) as a white solid.

^{1}H NMR ($CDCl_3$, 400 MHz, ppm): δ 8.63 (s, 4H, ArH), 8.10 (s, 4H, ArH), 1.47 ppm (s, 24H, CH_3). ^{13}C NMR ($CDCl_3$, 101 MHz, ppm): δ 131.24, 130.92, 127.67, 126.35, 84.20, 25.03. MALDI-TOF-MS, m/z: calculated for $C_{28}H_{32}B_2O_4$: 454.180; found, 453.977.

General procedure for preparation of (4a). The compound of **2a** (0.40 g, 1.34 mmol), **3** (0.30 g, 0.66 mmol) and $NaHCO_3$ (4.50 g, 53.57 mmol) were added to reaction tube. The mixture of purified THF (20 mL) and H_2O (9 mL) was added into the tube to dissolve reactants, subjected to three freeze-pump-thaw cycles and backfilled with a N_2 atmosphere. The catalyst $Pd(PPh_3)_4$ (0.04 mg, 0.03 mmol) was added under the N_2 atmosphere quickly and stirred at 80 °C for one day. Then, the reaction was quenched with water (100 mL) and extracted with CH_2Cl_2 (2 × 100 mL), and the combined organic extracts were washed with abundant water. The organic layer was dried over anhydrous $MgSO_4$ and concentrated under reduced pressure. The residue was purified by chromatography on a silica gel column, eluting with CH_2Cl_2/EA (v/v, 50/1) to afford **4a** as a white powder (0.22 g, 52%).

^{1}H NMR ($CDCl_3$, 400 MHz, ppm): δ 8.44 (d, *J* = 8.4 Hz, 2H, ArH), 8.25 (s, 4H, PyH), 8.16 (s, 4H, PyH), 7.49 (m, *J* = 7.5 Hz, 4H, ArH), 7.30 (t, *J* = 7.3 Hz, 2H, ArH), 7.21 (s, 2H, NH), 1.74 (s, 2H, CH_2), 1.55 (m, 4H, CH_2), 1.37-1.14 (m, 12H, CH_2), 0.86 (t, 6H, CH_3), 0.79 (t, 6H, CH_3). ^{13}C NMR ($CDCl_3$, 101 MHz, ppm): δ 174.25, 136.26, 135.05, 132.26, 131.66, 130.73, 128.82, 128.04, 126.16, 124.46, 123.79, 121.97, 50.95, 32.56, 29.86, 26.13, 22.72, 13.93, 12.14. MALDI-TOF-MS, m/z: calculated for $C_{44}H_{48}N_2O_2$ $[M+Na]^+$: 659.870; found, 659.519.

General procedure for preparation of (4b). According to the procedure for **4a,** the reaction mixture of **2b** (0.75 g, 2.52 mmol), **3** (0.54 g, 1.20 mmol) and $NaHCO_3$ (8.15 g, 97.01 mmol), THF (30 mL), H_2O (13 mL) and $Pd(PPh_3)_4$ (0.07 mg, 0.06 mmol). The reaction temperature, reaction time and after-treatment were as same as the procedure for **4a**. The residue was purified by chromatography on silica gel column eluting with PE/DCM (v/v, 2/1) to afford **4b** as a white powder (0.44 g, 58%).

^{1}H NMR ($CDCl_3$, 400 MHz, ppm): δ 8.40 (d, *J* = 8.4 Hz, 2H, ArH), 8.24 (s, 4H, PyH), 8.17 (s, 4H, PyH), 7.48 (m, *J* = 7.5 Hz, 4H, ArH), 7.29 (t, *J* = 7.3 Hz, ArH), 7.24 (s, 2H, NH), 2.11 (t, 4H, CH_2), 1.50 (t, 4H, CH_2), 1.14-1.09 (m, 16H, CH_2), 0.76 (t, 6H, CH_3). ^{13}C NMR ($CDCl_3$, 101 MHz, ppm): δ 171.38, 136.31, 135.08, 132.32, 131.70, 130.73, 128.83, 128.08, 126.05, 124.49, 123.76, 121.99, 37.91, 31.51, 29.07, 28.93, 25.55, 22.53, 13.98. MALDI-TOF-MS, m/z: calculated for $C_{44}H_{48}N_2O_2$ $[M+Na]^+$: 659.870; found, 659.628.

General procedure for preparation of (1,8-DNPy-B and 1,6-DNPy-B). A solution of **4a** (0.30 g, 0.47 mmol), P_2O_5 (0.54 g, 3.80 mmol), and $POCl_3$ (20 mL) was stirred at 125 °C for 30 h with a N_2

atmosphere. After cooling to room temperature, the reaction solution was added carefully to the ice water mixture. The aqueous phase was adjusted to pH = 9 with KOH solution and extracted with dichloromethane, then dried over anhydrous $MgSO_4$ and evaporated to dryness. The residue was purified by flash chromatography on a silica gel column, eluting with CH_2Cl_2/PE (v/v, 2/1) to afford a yellow powder (0.24 g, 85%). The isomers were separated by plate TLC (CH_2Cl_2/hexane = 1/1) or silica gel columns ($R_{f\ (\mathbf{1,8\text{-}DNPy\text{-}B})}$ = 0.4, $R_{f\ (\mathbf{1,6\text{-}DNPy\text{-}B})}$ = 0.5 in 1:1 v/v CH_2Cl_2/hexane, eluent: CH_2Cl_2/hexane = 1/3 to 5/1) to afford two isomers: **1,8-DNPy-B** (200 mg, 71%, green powder) and **1,6-DNPy-B** (34 mg, 12%, yellow powder).

14,17-di(heptan-3-yl)naphtho[1,8-*ij*:4,5-*i'j'*]diphenanthridine (1,8-DNPy-B).
^{1}H NMR ($CDCl_3$, 400 MHz, ppm): δ 9.19 (d, *J* = 9.2 Hz, 2H, ArH), 8.89-8.83 (m, 4H, PyH), 8.27 (d, *J* = 8.1 Hz, 2H, ArH), 8.12 (d, *J* = 8.3 Hz, 2H, PyH), 7.84 (t, *J* = 7.8 Hz, 2H, ArH), 7.74 (t, *J* = 7.7 Hz, 2H, ArH), 4.39 (m, *J* = 4.3 Hz, 2H, CH), 2.29 (m, 4H, CH_2), 2.14 (m, 4H, CH_2), 1.30-1.22 (m, 8H, CH_2), 0.80 (m, 12H, CH_3). ^{13}C NMR (101 MHz, $CDCl_3$) δ 164.90, 143.98, 132.31, 129.49, 129.44, 128.77, 127.07, 126.04, 125.36, 125.31, 122.39, 122.36, 121.33, 119.67, 119.63, 47.69, 35.25, 29.99, 28.61, 22.88, 13.96, 12.33. HR-ESI-MS, m/z: calculated for $C_{44}H_{44}N_2$ $[M+H]^+$, 601.3584; found, 601.3575.

6,15-di(heptan-3-yl)naphtho[1,8-*ij*:5,4-*i'j'*]diphenanthridine (1,6-DNPy-B).
^{1}H NMR ($CDCl_3$, 400 MHz, ppm): δ 9.43 (s, 2H, PyH), 9.02 (d, *J* = 9.0 Hz, 2H, ArH), 8.87 (d, *J* = 8.8 Hz, 2H, PyH), 8.47 (d, *J* = 8.4 Hz, 2H, ArH), 8.24 (d, *J* = 8.2 Hz, 2H, PyH), 7.82 (t, *J* = 7.8 Hz, 2H, ArH), 7.74 (t, *J* = 7.7 Hz, 2H, ArH), 4.18 (m, J = 4.2 Hz, 2H, CH), 2.26 (m, 4H, CH_2), 2.10 (m, 4H, CH_2), 1.25 (m, 8H, CH_2), 0.88-0.77 (m, 12H, CH_3). ^{13}C NMR ($CDCl_3$, 101 MHz, ppm): δ 165.47, 143.49, 131.09, 130.90, 129.29, 128.74, 128.16, 127.28, 126.15, 124.61, 122.47, 122.37, 121.64, 119.42, 99.99, 48.35, 35.34, 29.99, 28.78, 22.94, 13.97, 12.30. HR-ESI-MS, m/z: calculated for $C_{44}H_{44}N_2$ $[M+H]^+$, 601.3584; found, 601.3574.

General procedure for preparation of (1,8-DNPy-L and 1,6-DNPy-L). It was synthesized following the procedure above, with a solution of **4b** (0.30 g, 0.47 mmol), P_2O_5 (0.50 g, 3.52 mmol), and $POCl_3$ (20 mL). After the reaction was completed, the post-treatment method was as same as above, the residue was purified by flash chromatography on a silica gel column, eluting with CH_2Cl_2 to afford a yellow-green powder (0.24 g, 85%). The isomers were separated by plate TLC (CH_2Cl_2/hexane = 2/1) or silica gel columns ($R_{f\ (\mathbf{1,8\text{-}DNPy\text{-}B})}$ = 0.5, $R_{f\ (\mathbf{1,6\text{-}DNPy\text{-}B})}$ = 0.6 in 5:1 v/v CH_2Cl_2/hexane, eluent: CH_2Cl_2/hexane = 2/1 to 25/1) to afford two isomers: **1,8-DNPy-L** (186 mg, 66%, green powder) and **1,6-DNPy-L** (46 mg, 16%, yellow powder).

14,17-diheptylnaphtho[1,8-*ij*:4,5-*i'j'*]diphenanthridine (1,8-DNPy-L).
^{1}H NMR ($CDCl_3$, 400 MHz, ppm): δ 9.14 (d, *J* = 9.1 Hz, 4H, PyH), 8.78 (d, *J* = 8.8 Hz, 2H, ArH), 8.25 (d, *J* = 8.2 Hz, 2H, ArH), 8.09 (s, 2H, PyH), 7.81 (t, *J* = 7.8 Hz, 2H, ArH), 7.71 (t, *J* = 7.7 Hz, 2H, ArH), 3.39 (m, *J* = 3.9 Hz, 4H, CH_2), 2.20 (m, 4H, CH_2), 1.62 (m, 4H, CH_2), 1.48 (m, 4H, CH_2), 1.35 (m, 8H, CH_2), 0.90 (t, 6H, CH_3). ^{13}C NMR ($CDCl_3$, 101 MHz, ppm): δ 161.00, 143.38, 132.31, 132.04, 129.55, 129.09, 128.93, 127.36, 126.30, 125.58, 124.20, 122.73, 122.31, 120.56, 119.89, 41.97, 31.97, 30.01, 29.59, 29.35, 22.73, 14.16. HR-ESI-MS, m/z: calculated for $C_{44}H_{44}N_2$ $[M+H]^+$, 601.3584; found, 601.3577.

6,15-diheptylnaphtho[1,8-*ij*:5,4-*i'j'*]diphenanthridine (1,6-DNPy-L).

^{1}H NMR ($CDCl_3$, 400 MHz, ppm): δ 9.41 (s, 2H, PyH), 9.15 (d, J = 9.1 Hz, 2H, ArH), 8.87 (d, J = 8.8 Hz, 2H, PyH), 8.45 (d, J = 8.4 Hz, 2H, ArH), 8.24 (d, J = 8.2 Hz, 2H, PyH), 7.84 (t, J = 7.8 Hz, 2H, ArH), 7.77 (t, J = 7.7 Hz, 2H, ArH), 3.86 (m, J = 3.8 Hz, 4H, CH_2), 2.17 (m, 4H, CH_2), 1.55 (m, 4H, CH_2), 1.45 (m, 4H, CH_2), 1.33 (m, 8H, CH_2), 0.90 (m, 6H, CH_3). ^{13}C NMR ($CDCl_3$, 101 MHz, ppm): δ161.52, 142.90, 131.42, 130.98, 129.03, 128.95, 128.57, 127.78, 126.49, 124.27, 122.79, 122.47, 120.97, 119.71, 42.35, 31.87, 29.89, 29.62, 29.25, 22.67, 14.13. HR-ESI-MS, m/z: calculated for $C_{44}H_{44}N_2$ $[M+H]^+$, 601.3584; found, 601.3574.

Preparation of single crystals for singel crystal X-ray measurements: Single crystals of **1,8-DNPy-B** and **1,6-DNPy-B** suitable for X-ray measurements were obtained by slow evaporation of chloroform/isopropyl alcohol solution.

Results and Discussion

The mixture of **1,8-DNPy-L** and **1,6-DNPy-L** tended to form self-assembled organogels in various aromatic solvents such as toluene and chlorobenzene. In contrast, **1,8-DNPy-L** and **1,6-DNPy-L** alone could not form gels in organic solvents. This finding indicated the existence of interactions between **1,8-DNPys** and **1,6-DNPys**:

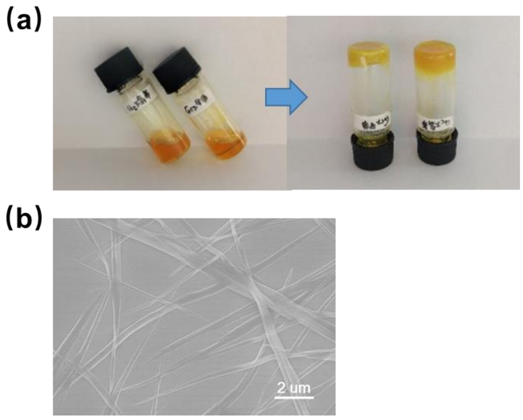

Figure S1. (a) Photographs showing gel formation from the mixture of **1,8-DNPy-L** and **1,6-DNPy-L** in chlorobenzene (left) and toluene (right). (b) SEM image of self-assembled gel of the mixture of **1,8-DNPy-L** and **1,6-DNPy-L** in chlorobenzene.

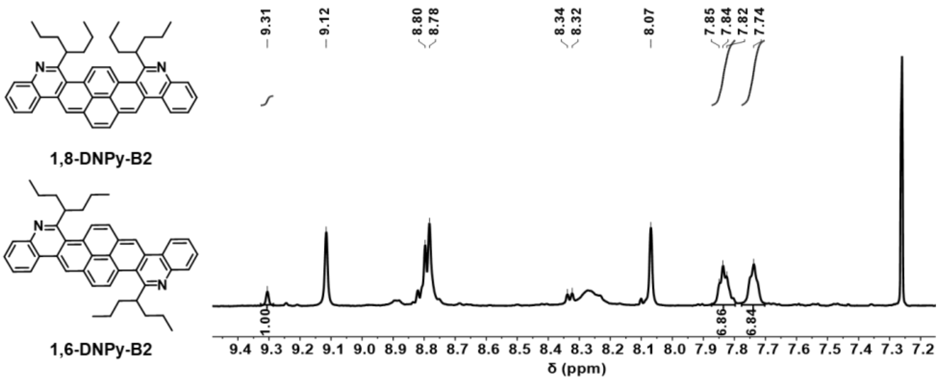

Figure S2. Chemical structures of **1,8-DNPy-B2** and **1,6-DNPy-B2** and partial ^{1}H NMR spectrum of the as-prepared mixture of **1,8-DNPy-B2** and **1,6-DNPy-B2**.

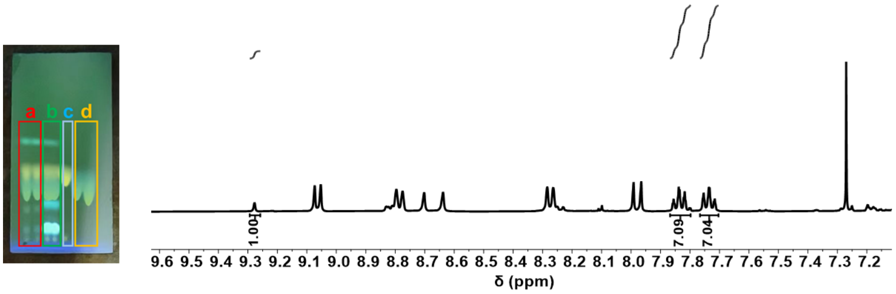

Figure S3. Left: thin layer chromatography of the cyclization reaction of **4a** (a corresponds to the reaction mixture using toluene as the solvent, b corresponds to the one with CCl_4 as the solvent, c corresponds to pure **1,6-DNPy-B**, and d corresponds to pure **1,8-DNPy-B**). Right: partial ^{1}H NMR spectrum of the as-synthesized crude product using toluene as the solvent.

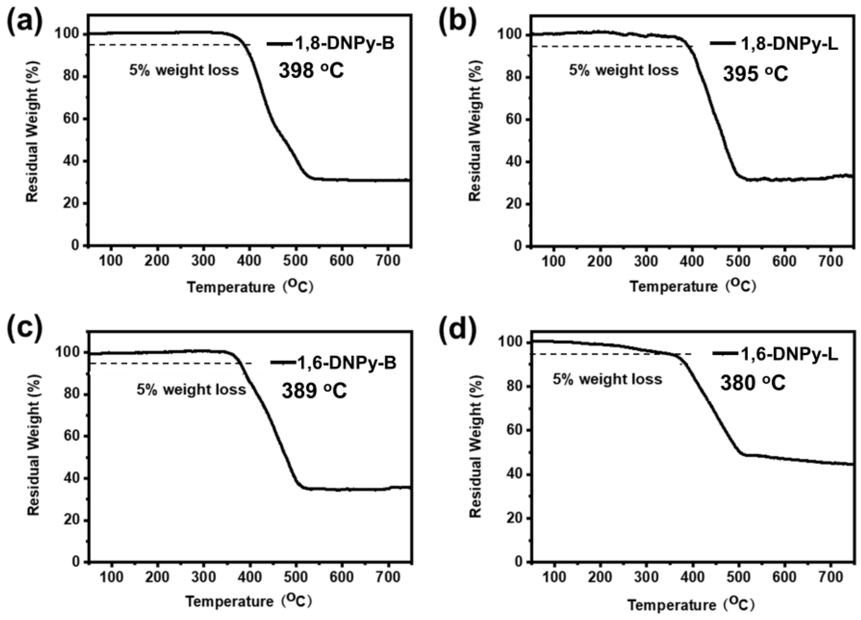

Figure S4. TGA traces of (a) **1,8-DNPy-B** (b) **1,8-DNPy-L** (c) **1,6-DNPy-B** and (d) **1,6-DNPy-L** at a rate of 10 °C/min, the 5% weight loss temperature has been marked in the figure.

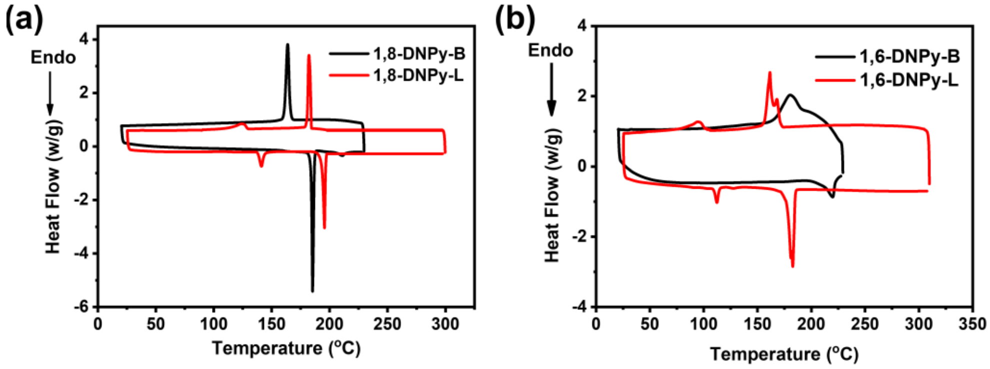

Figure S5. DSC traces of (a) **1,8-DNPy-B** and **1,8-DNPy-L** (b) **1,6-DNPy-B** and **1,6-DNPy-L** at a rate of 10 °C/min.

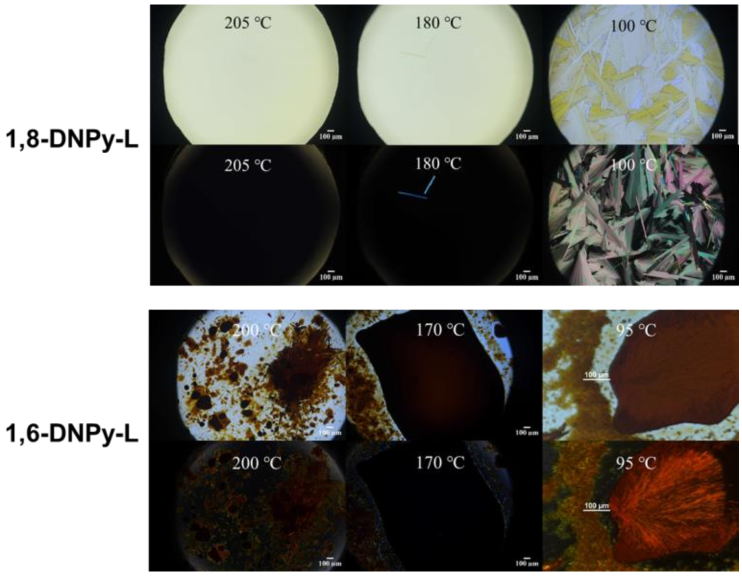

Figure S6. Optical microscopic images of **1,8-DNPy-L** (top) and **1,6-DNPy-L** (bottom) under different temperatures (cooling rate: 2 ℃/min, the upper images are without polarizer, and the lower images are with polarizer).

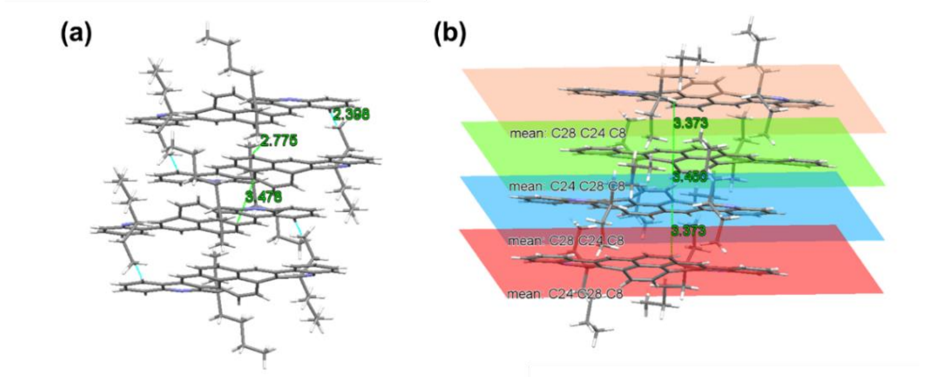

Figure S7. Multiple intermolecular interactions of (a) **1,8-DNPy-B** existed in the crystals, including π···π, C-H···C(CH_3)-H and C-H···π interactions. (b) The distance between the rigid planes of two adjacent molecules of **1,8-DNPy-B**.

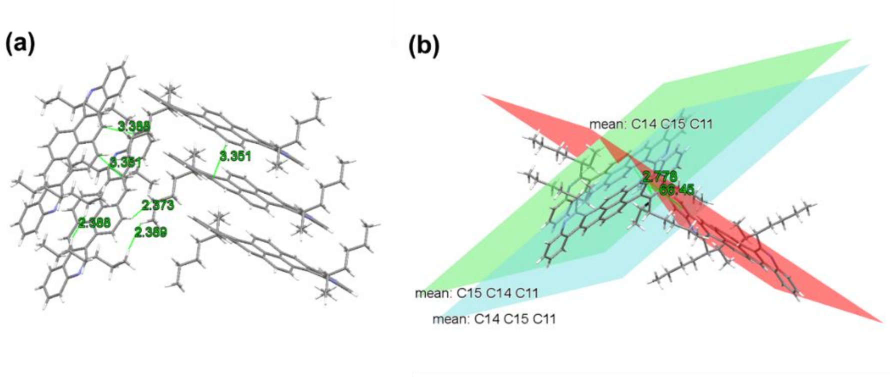

Figure S8. Multiple intermolecular interactions of (a) **1,6-DNPy-B** existed in the crystals, including π···π, C(CH_3)-H···C(CH_3)-H, C(CH_2)-H···C(CH_3)-H, C···C and C-H···π interactions. (b) The distance and angle between the rigid planes of two adjacent molecules of **1,6-DNPy-B**.

Table S1. Crystal data of **1,8-DNPy-B** (CCDC: 2180019).

Experical formula	C44 H44 N2
Space group	P -1
Cell lengths	a/Å 7.4324(5) b/Å 14.6018(14) c/Å 16.6369(14)
Cell angles	α/° 109.684(8) β/° 98.078(7) γ/° 93.683(7)
Cell volume	1671.07/$Å^3$
Z, Z’	Z: 2 Z': 0
R-Factor (%)	8.87

Table S2. Crystal data of **1,6-DNPy-B** (CCDC: 2180027).

Experical formula	C44 H44 N2
Space group	P 2_1/n
Cell lengths	a/Å 5.43800(10) b/Å 17.7484(5) c/Å 19.3921(6)
Cell angles	α/° 90 β/° 91.455(2) γ/° 90
Cell volume	1871.04/$Å^3$
Z, Z’	Z: 4 Z': 0
R-Factor (%)	8.14

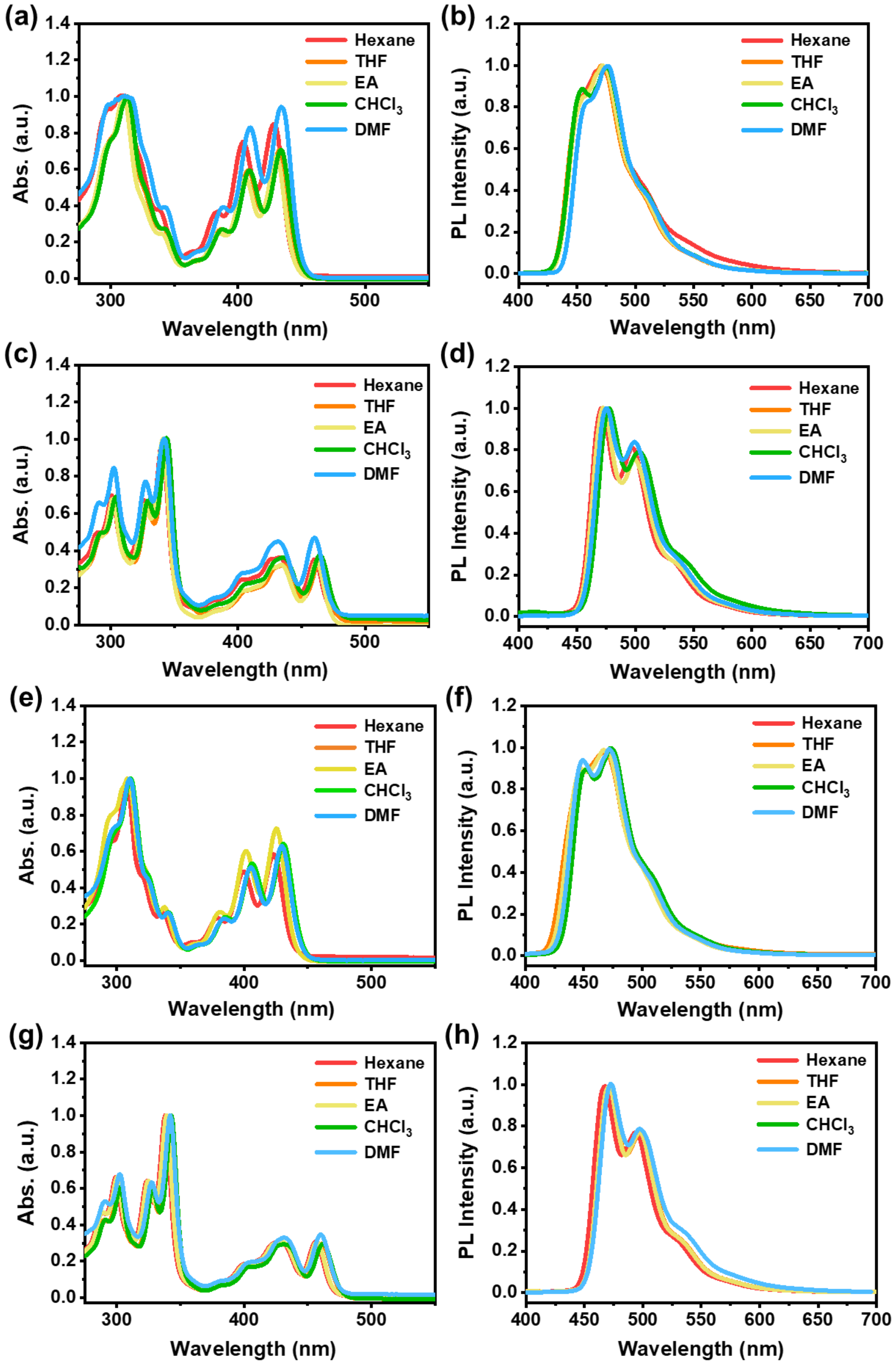

Figure S9. (a, c, e, g) UV-vis absorption and (b, d, f, h) fluorescence spectra of **1,8-DNPy-B** (a, b); **1,6-DNPy-B** (c, d); **1,8-DNPy-L** (e, f) and **1,6-DNPy-L** (g, h) in different solvents (1×10^{-5} M, λ_{ex} = 410 nm for **1,8-DNPys**, and λ_{ex} = 429 nm for **1,6-DNPys**).

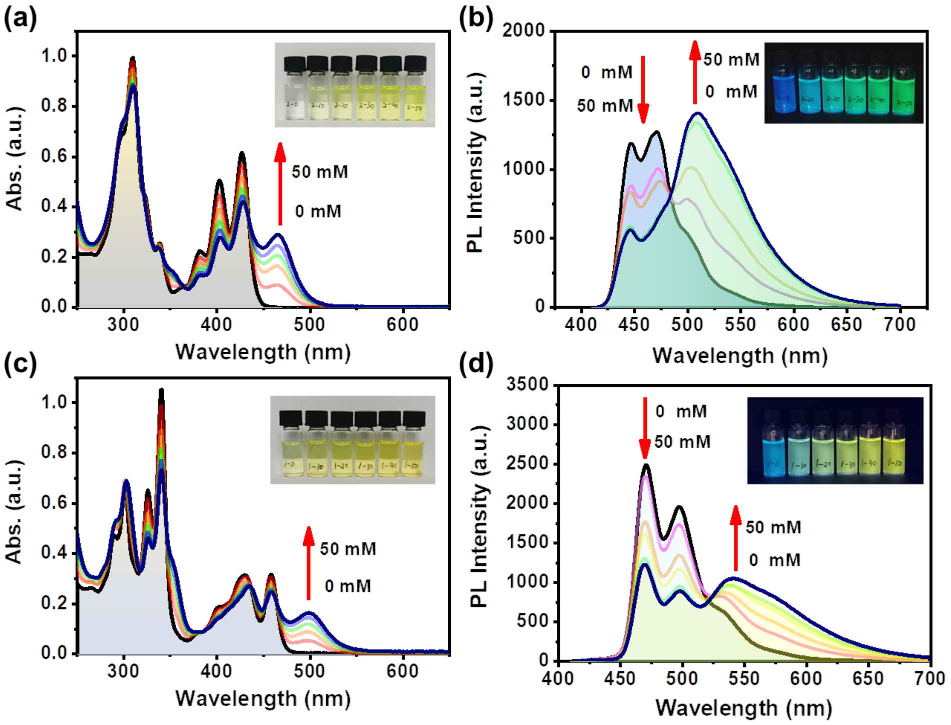

Figure S10. (a) UV-vis absorption spectra, (b) fluorescence spectra of **1,8-DNPy-B**/**1,8-DNPy-L** in dichloromethane with different concentrations of TFA. (c) UV–vis absorption spectra, (d) fluorescence spectra of **1,6-DNPy-B**/**1,6-DNPy-L** in dichloromethane with different concentrations of TFA.

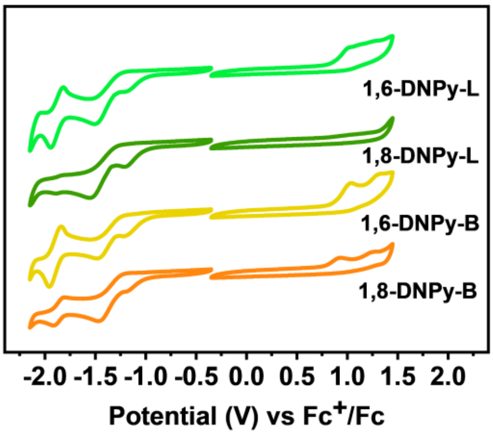

Figure S11. Cyclic voltammograms of **1,8-DNPy-B**, **1,6-DNPy-B**, **1,8-DNPy-L** and **1,6-DNPy-L** measured in CH_2Cl_2 (1 mM) with 0.1 M $TBAPF_6$ as supporting electrolyte (scan rate = 100 mVs^{-1}).

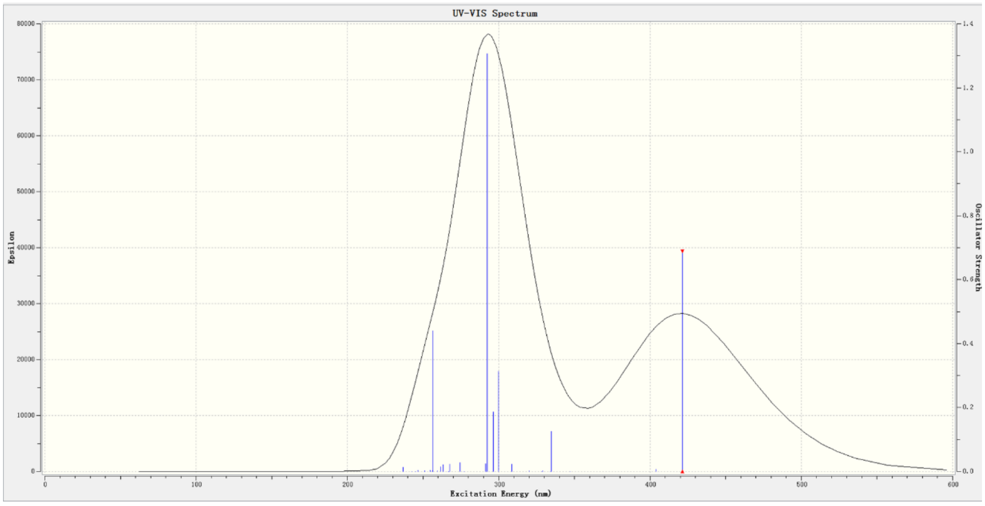

Figure S12. Simulated UV-vis absorption spectrum of **1,8-DNPy-L** in vacuo by TD-DFT calculation.

Table S3. Main orbital transitions of **1,8-DNPy-L** calculated with TD-DFT.

λ_{abs}[a] (nm)	λ_{abs}[b] (nm)	f [c]	Transition (%)[d]
427	421.29	0.6882	H-1→L+1 (4.7%)
			H→L (95.3%)
338	334.85	0.1253	H-1→L (28.8%)
			H→L+1 (71.2%)
310	299.80	0.3138	H-5→L (4.1%)
			H-3→L (6.9%)
			H-2→L+1 (3.2%)
			H-1→L+1 (22.5%)
			H-1→L+2 (13.1%)
			H→L+3 (47.6%)
			H→L+5 (2.6%)
	296.34	0.1864	H-7→L (68.2%)
			H-3→L (6.5%)
			H-1→L+1 (12.3%)
			H-1→L+2 (13.0%)
	292.33	1.3065	H-7→L (18.4%)
			H-2→L (3.5%)
			H-1→L+1 (56.6%)
			H-1→L+2 (6.5%)
			H→L (4.9%)
			H→L+1 (7.5%)
			H→L+5 (2.6%)

[a] Experimental absorption in THF; [b] Compound transition wavelength in vacuo; [c] Compound oscillator strength in vacuo; [d] H represents HOMO, L represents LUMO.

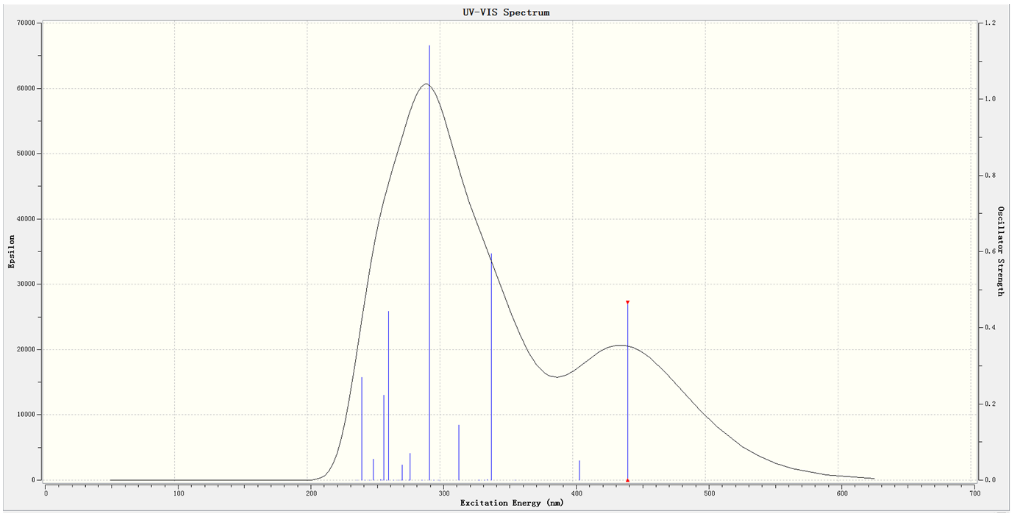

Figure S13. Simulated UV-vis absorption spectrum of **1,6-DNPy-L** in vacuo by TD-DFT calculation.

Table S4. Main orbital transitions of **1,6-DNPy-L** calculated with TD-DFT.

λ_{abs}[a] (nm)	λ_{abs}[b] (nm)	f [c]	Transition (%)[d]
460	441.5	0.4666	H-1→L+1 (3.3%)
			H→L (96.7%)
340	338.83	0.5939	H-4→L (8.6%)
			H-1→L (30.5%)
			H→L+1 (60.9%)
325	314.09	0.1440	H-4→L (85.7%)
			H→L+1 (5.8%)
			H→L+4 (8.5%)
301	291.99	1.1405	H-2→L+2 (2.5%)
			H-1→L+1 (94.2%)
			H→L (3.3%)

[a] Experimental absorption in THF; [b] Compound transition wavelength in vacuo; [c] Compound oscillator strength in vacuo; [d] H represents HOMO, L represents LUMO.

Table S5. DFT calculated molecular information of **1,8-DNPy-B**, **1,6-DNPy-B**, **1,8-DNPy-L** and **1,6-DNPy-L**.

	LUMO (eV)	HOMO (eV)	E_g (eV)	Dipole moment (Debye)
1,8-DNPy-B	-2.35	-5.39	3.04	2.20
1,6-DNPy-B	-2.41	-5.31	2.90	0.80
1,8-DNPy-L	-2.32	-5.41	3.09	2.49
1,6-DNPy-L	-2.40	-5.33	2.93	0.64

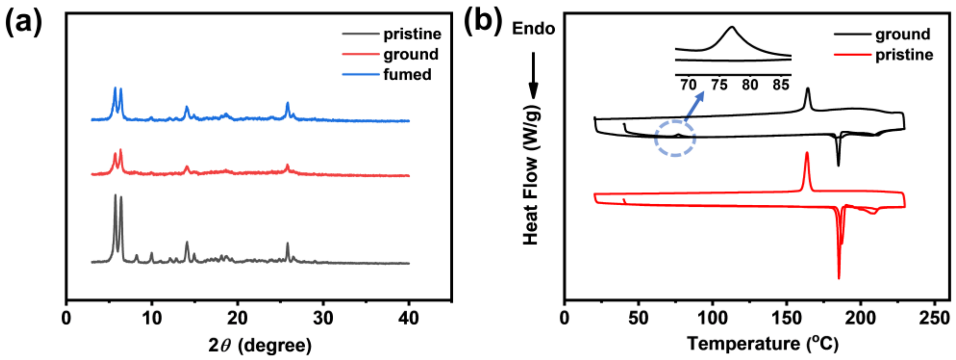

Figure S14. (a) PXRD patterns of **1,8-DNPy-B** in different solid states. The sample was fumed with CH_2Cl_2 vapor. (b) DSC traces of **1,8-DNPy-B** in different solid states. The illustration is an enlargement of the circled part.

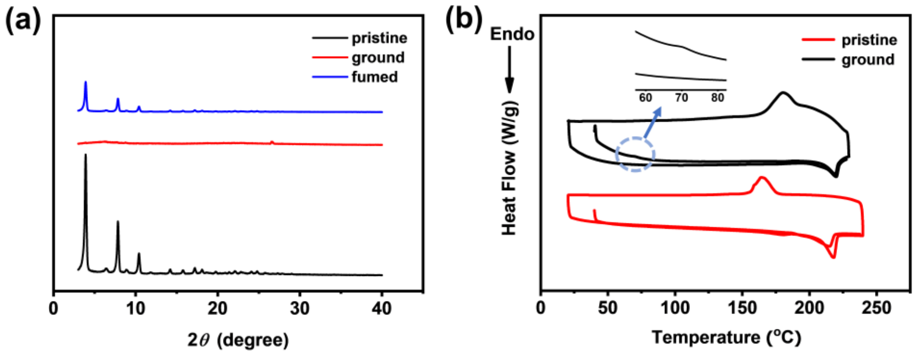

Figure S15. (a) PXRD patterns of **1,6-DNPy-B** in different solid states. The sample was fumed with CH_2Cl_2 vapor. (b) DSC traces of **1,6-DNPy-B** in different solid states. The illustration is an enlargement of the circled part.

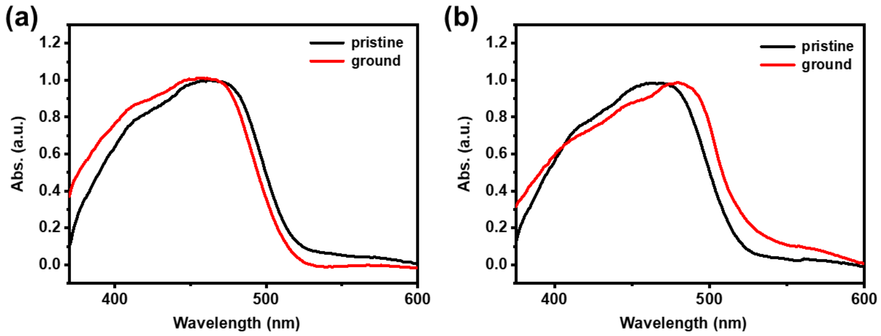

Figure S16. UV-vis absorption spectra of (a) **1,8-DNPy-B** and (b) **1,6-DNPy-B** in different solid states.

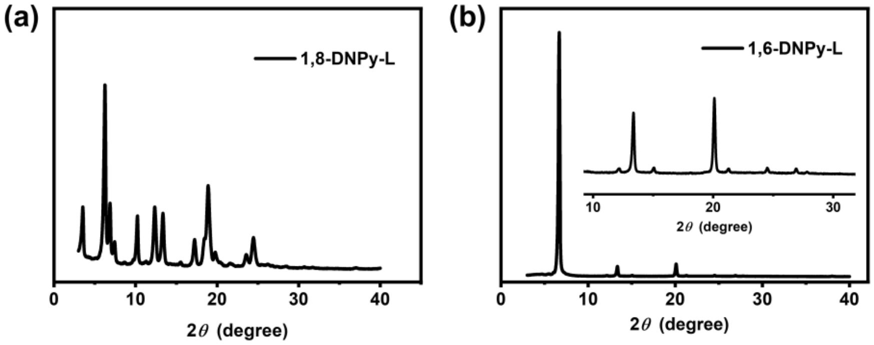

Figure S17. PXRD patterns of (a) **1,8-DNPy-L** and (b) **1,6-DNPy-L** in solid states.

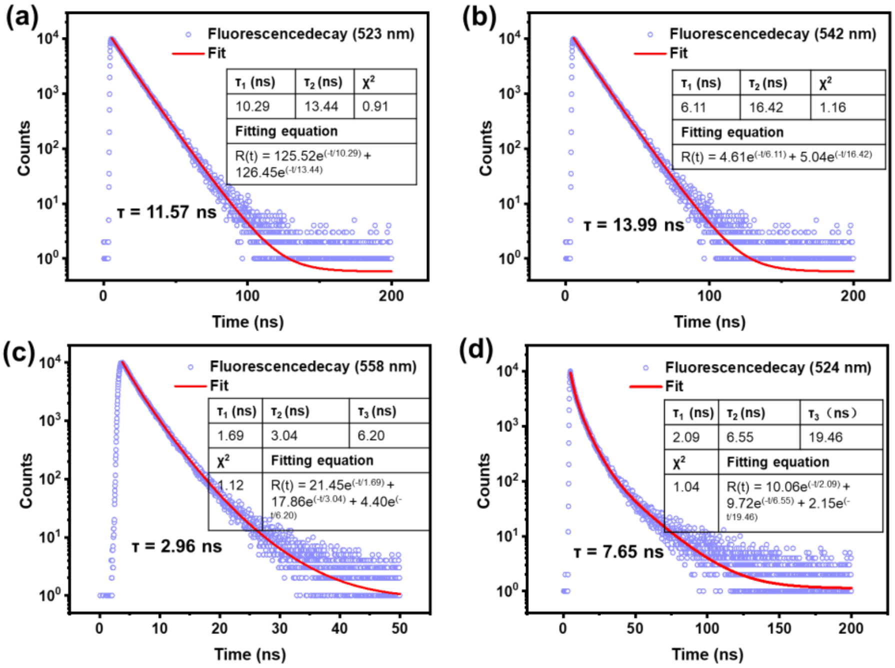

Figure S18. The fluorescence decay curves of **1,8-DNPy-B** (a) before and (b) after ground. The fluorescence decay curves of **1,6-DNPy-B** (c) before and (d) after ground at different emission wavelengths (λ_{ex} = 375 nm).

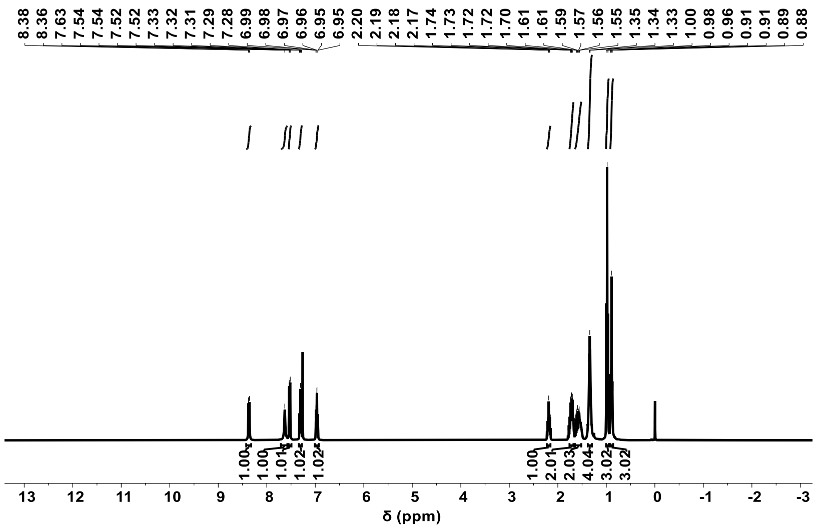

Figure S19. ^{1}H NMR spectrum of compound **2a** in $CDCl_3$.

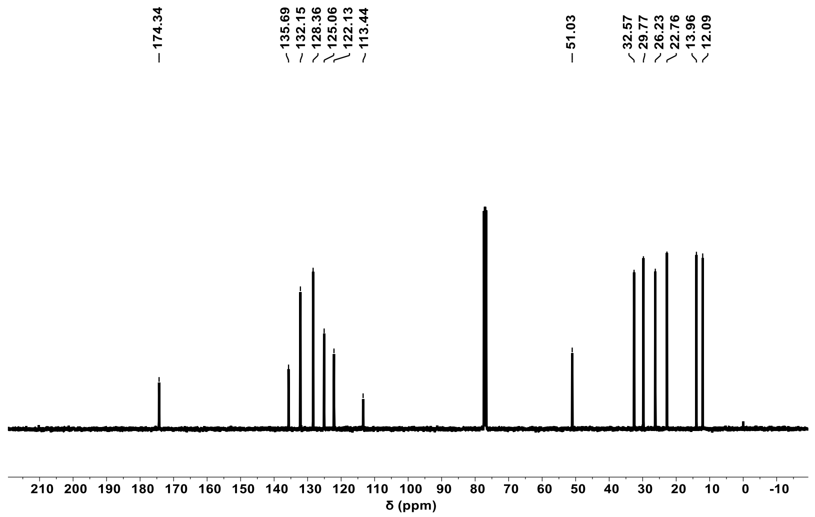

Figure S20. ^{13}C NMR spectrum of compound **2a** in $CDCl_3$.

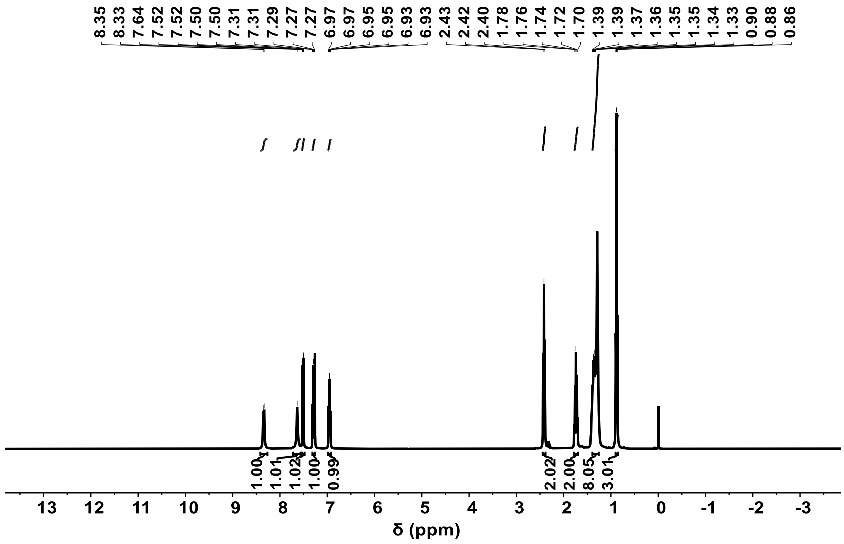

Figure S21. ^{1}H NMR spectrum of compound in **2b** $CDCl_3$.

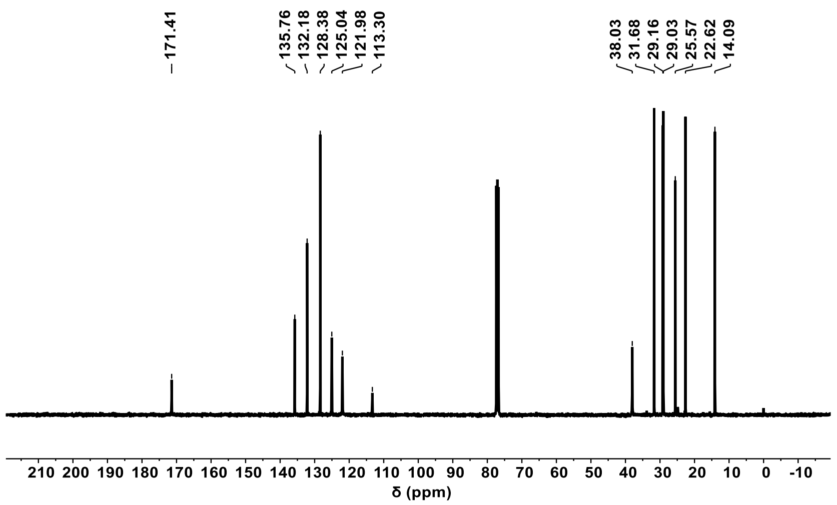

Figure S22. ^{13}C NMR spectrum of compound **2b** in $CDCl_3$.

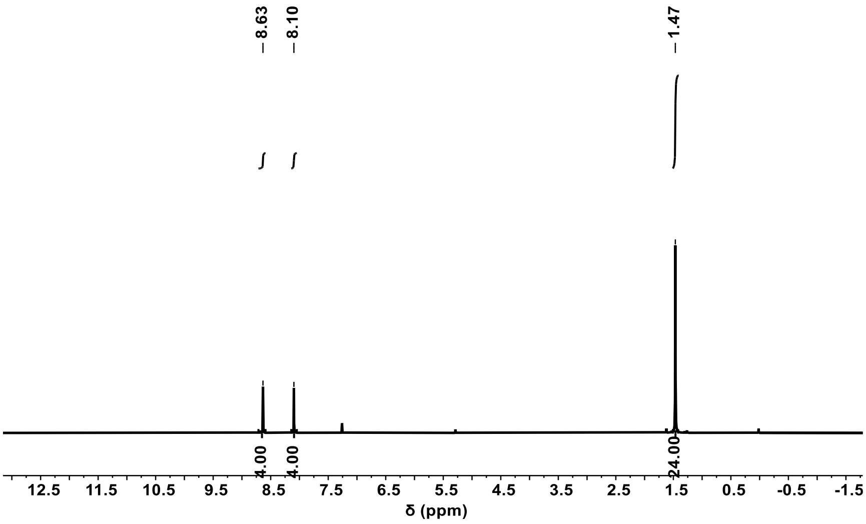

Figure S23. ^{1}H NMR spectrum of compound in **3** $CDCl_3$.

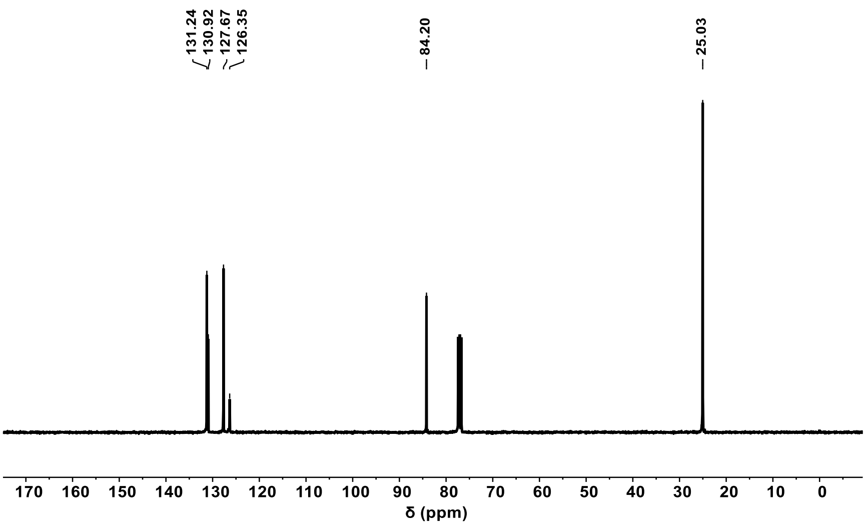

Figure S24. ^{13}C NMR spectrum of compound **3** in $CDCl_3$.

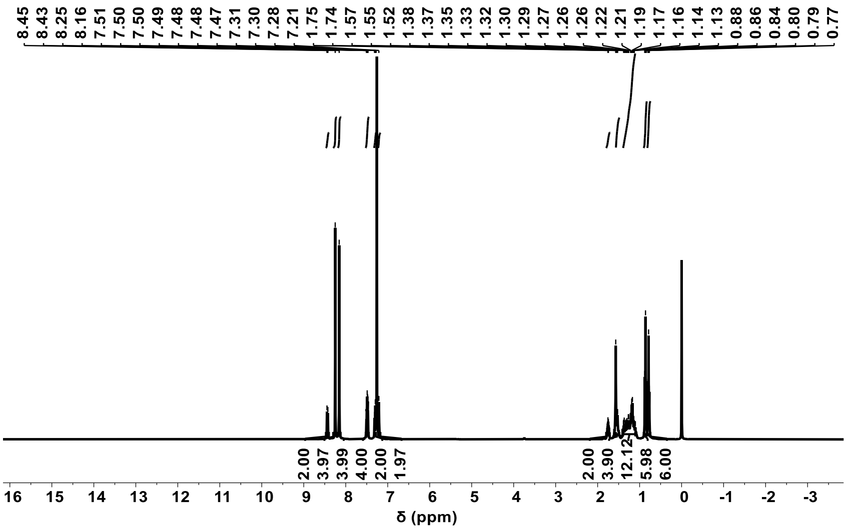

Figure S25. ^{1}H NMR spectrum of compound in **4a** $CDCl_3$.

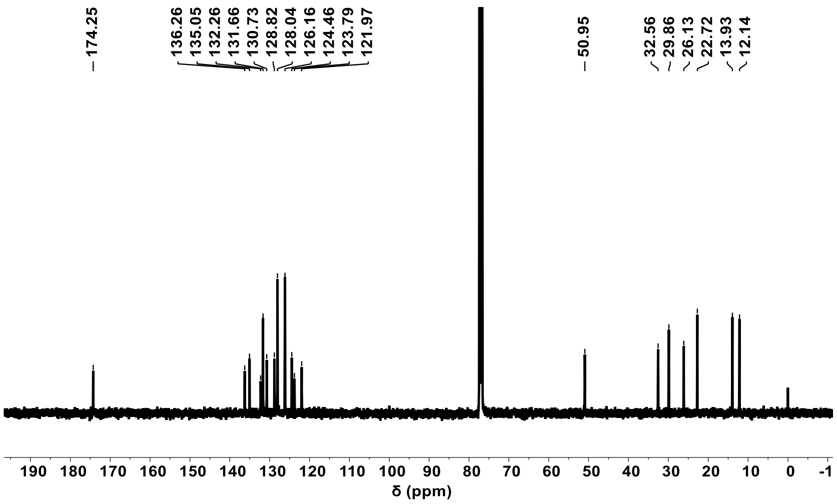

Figure S26. ^{13}C NMR spectrum of compound **4a** in $CDCl_3$.

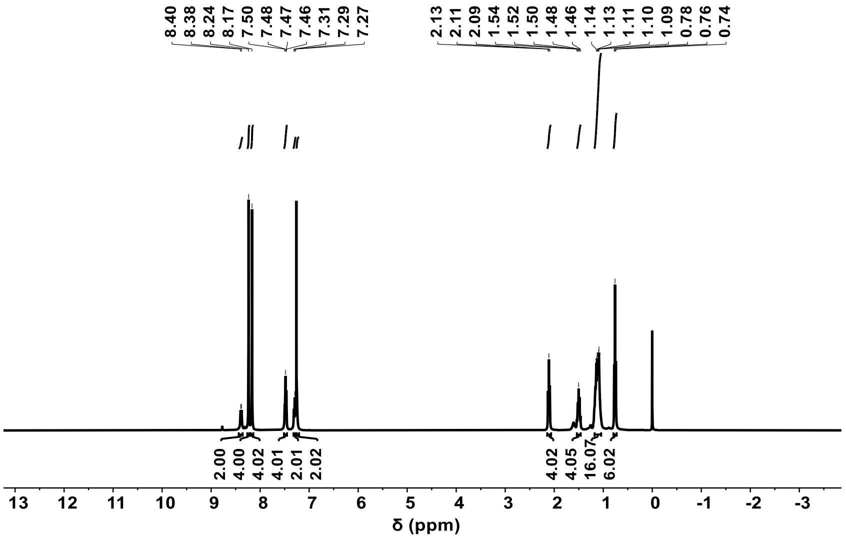

Figure S27. ^{1}H NMR spectrum of compound in **4b** $CDCl_3$.

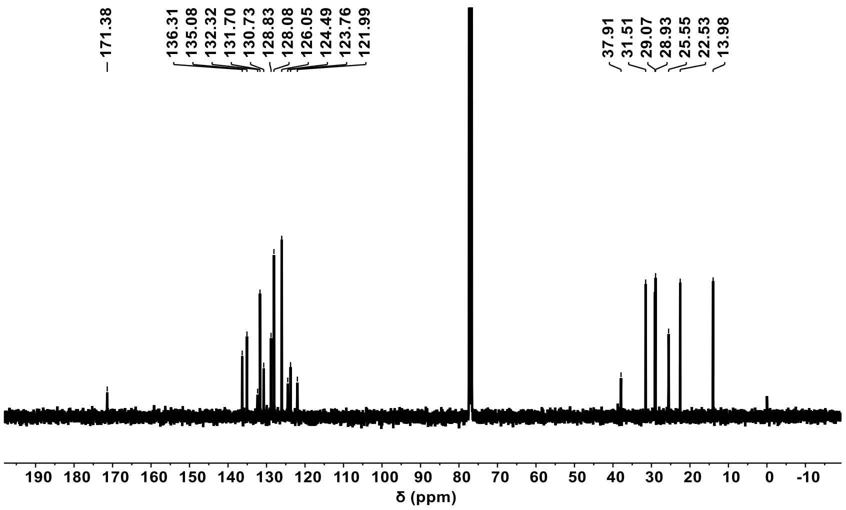

Figure S28. ^{13}C NMR spectrum of compound **4b** in $CDCl_3$.

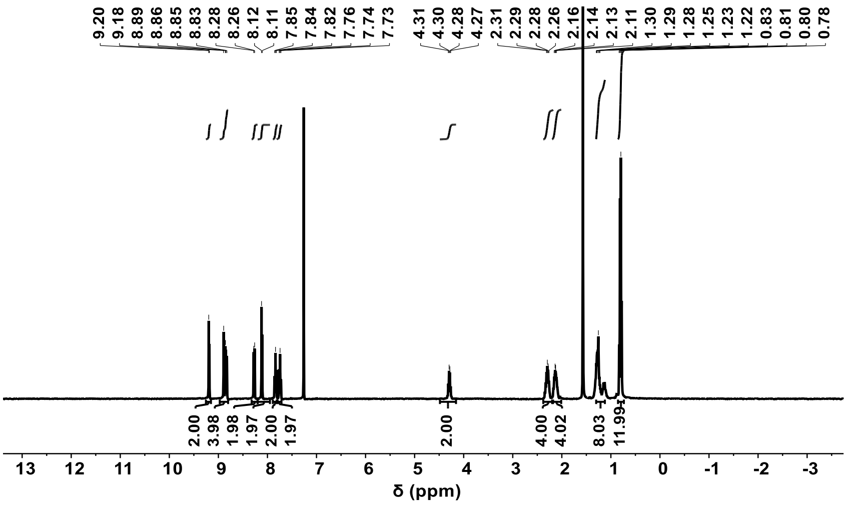

Figure S29. ^{1}H NMR spectrum of compound in **1,8-DNPy-B** $CDCl_3$.

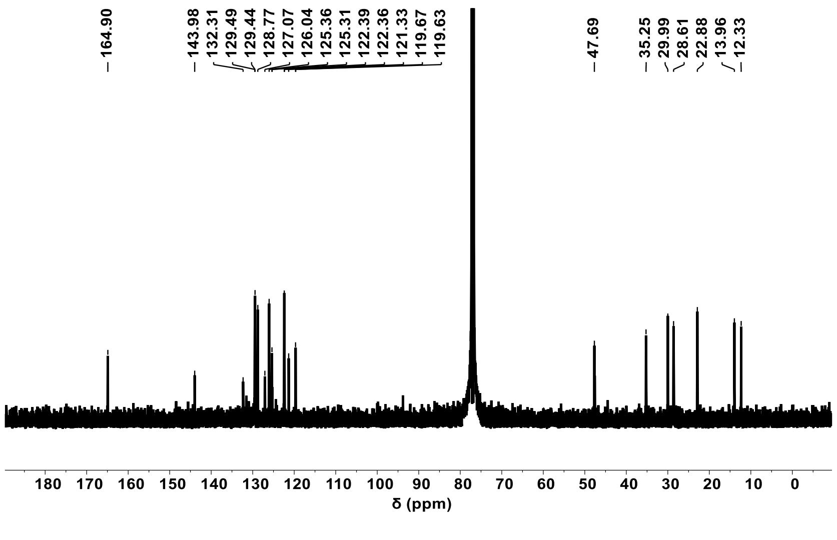

Figure S30. ^{13}C NMR spectrum of compound **1,8-DNPy-B** in $CDCl_3$.

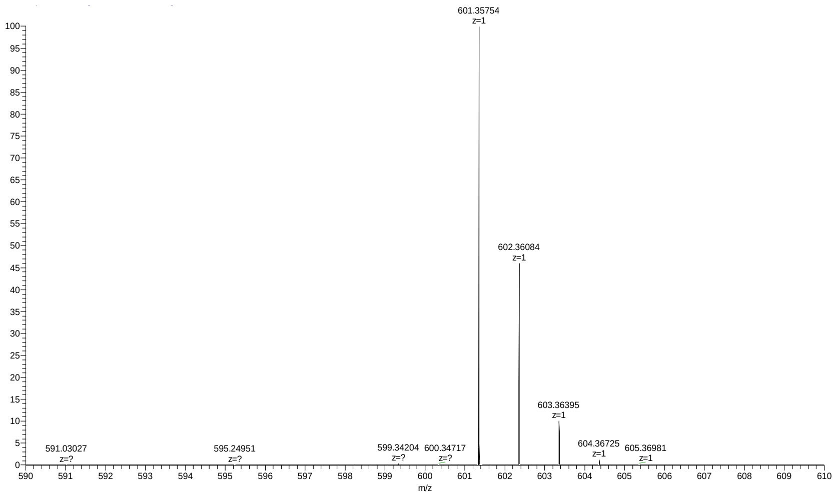

Figure S31. HR-ESI-MS spectrum of compound **1,8-DNPy-B.**

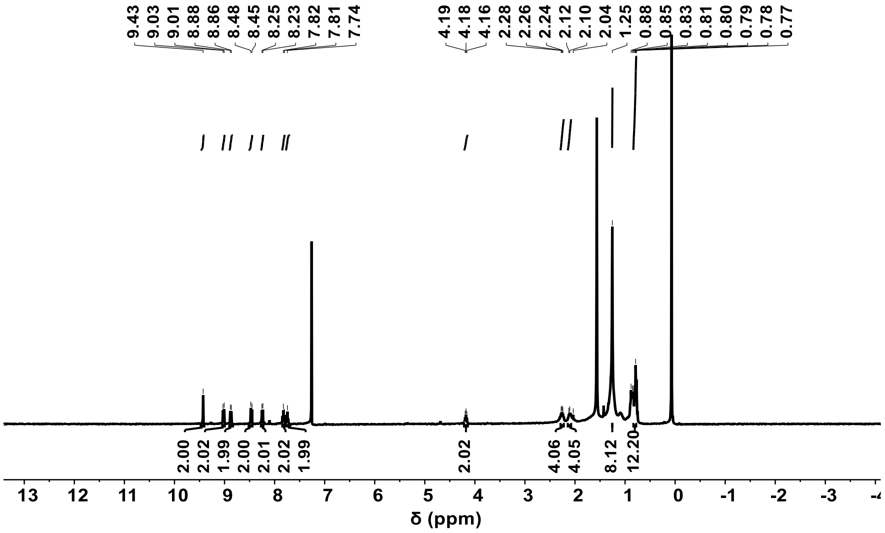

Figure S32. ^{1}H NMR spectrum of compound in **1,6-DNPy-B** $CDCl_3$.

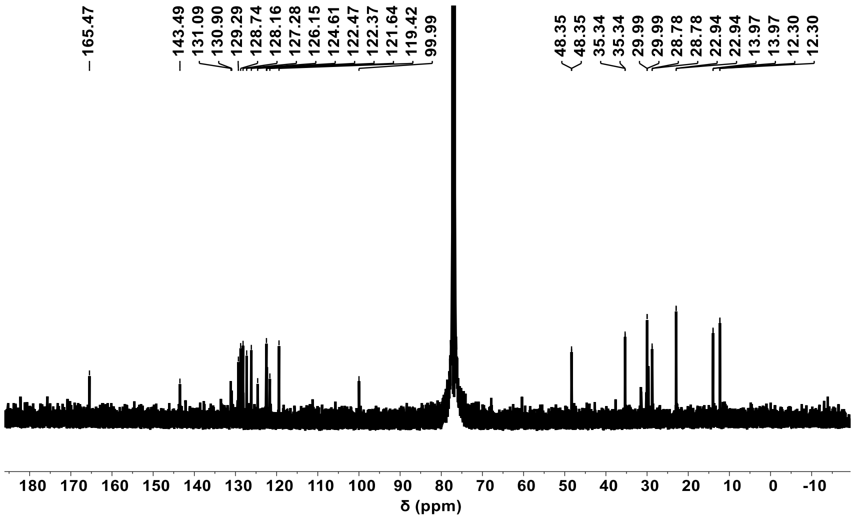

Figure S33. ^{13}C NMR spectrum of compound **1,6-DNPy-B** in $CDCl_3$.

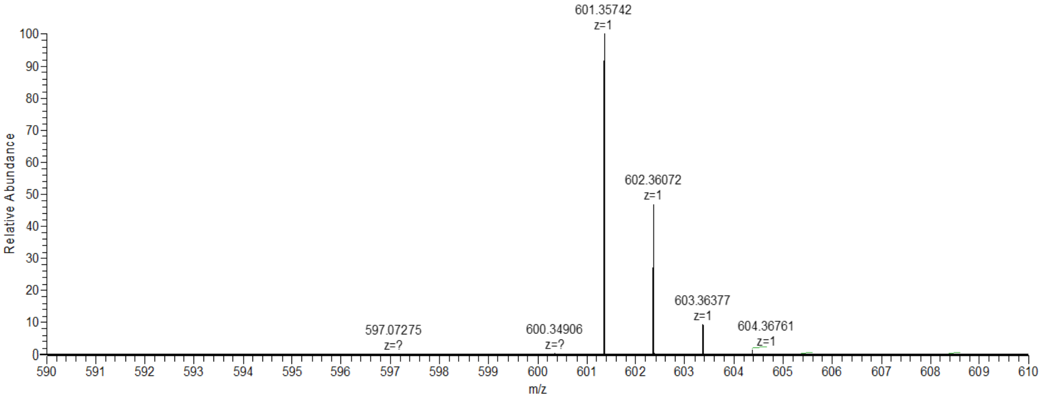

Figure S34. HR-ESI-MS spectrum of compound **1,6-DNPy-B.**

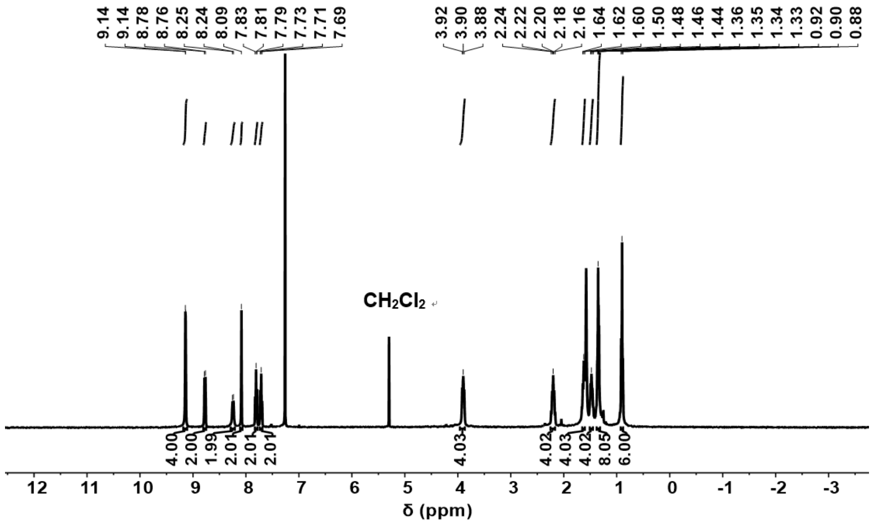

Figure S35. ^{1}H NMR spectrum of compound in **1,8-DNPy-L** $CDCl_3$.

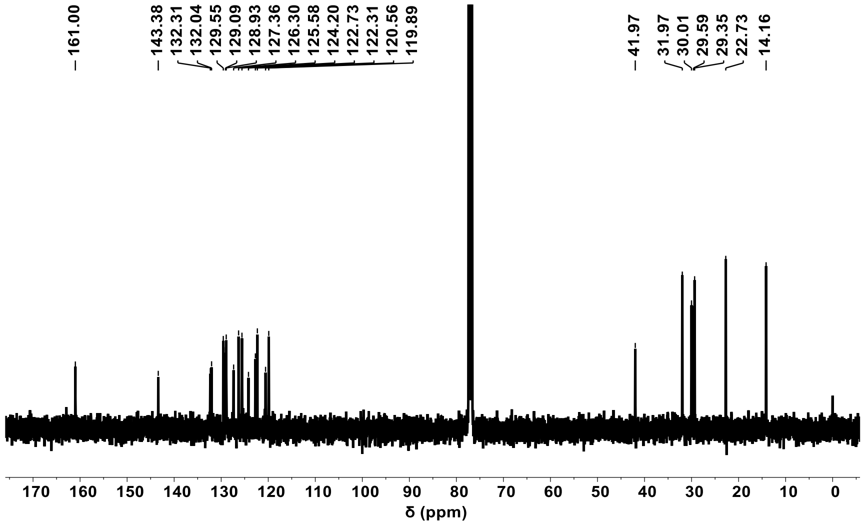

Figure S36. ^{13}C NMR spectrum of compound **1,8-DNPy-L** in $CDCl_3$.

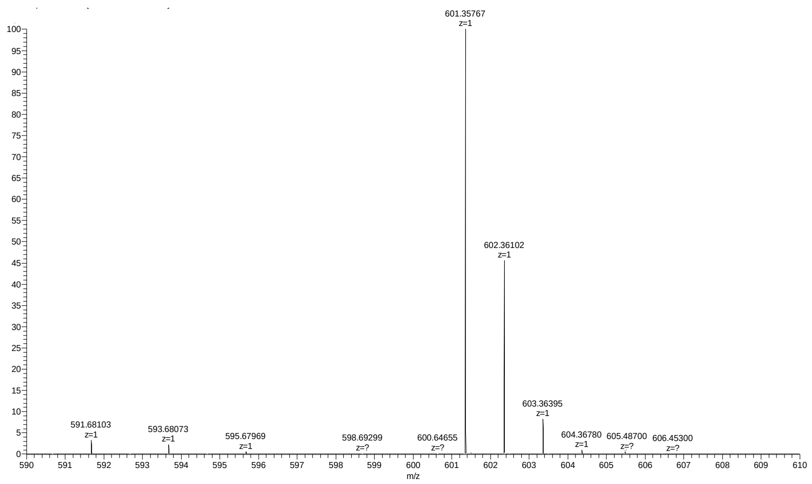

Figure S37. HR-ESI-MS spectrum of compound **1,8-DNPy-L**.

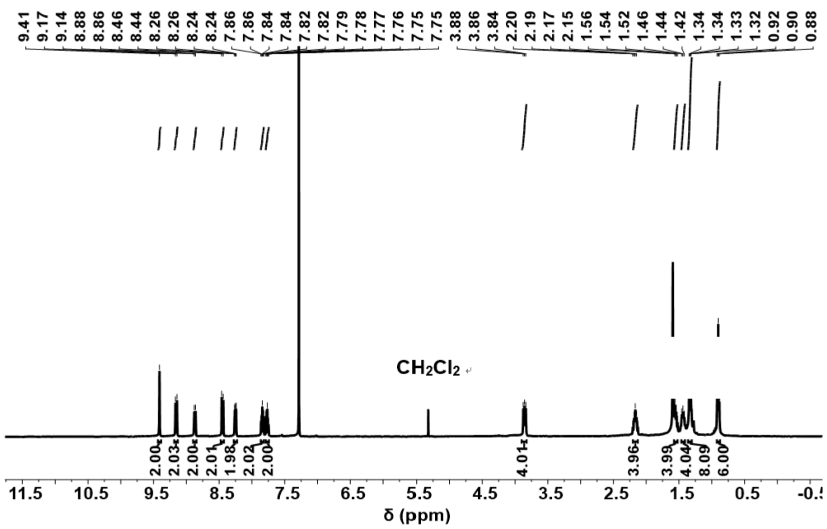

Figure S38. ^{1}H NMR spectrum of compound in **1,6-DNPy-L** $CDCl_3$.

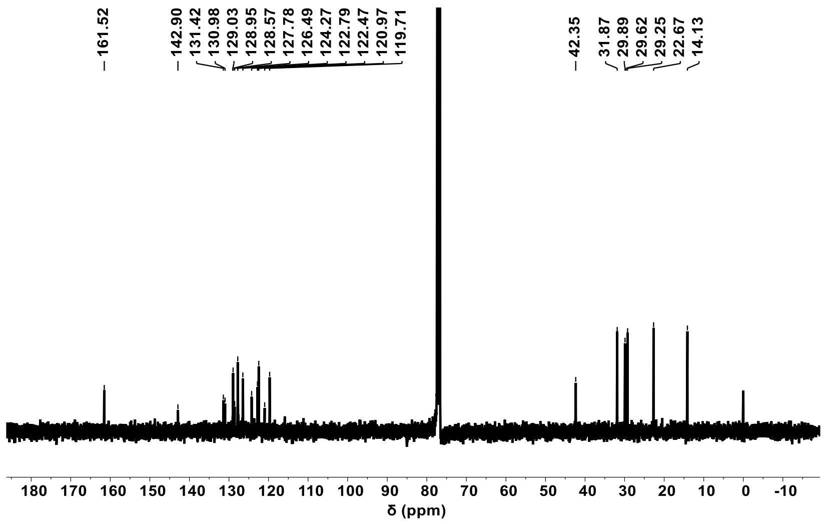

Figure S39. ^{13}C NMR spectrum of compound **1,6-DNPy-L** in $CDCl_3$.

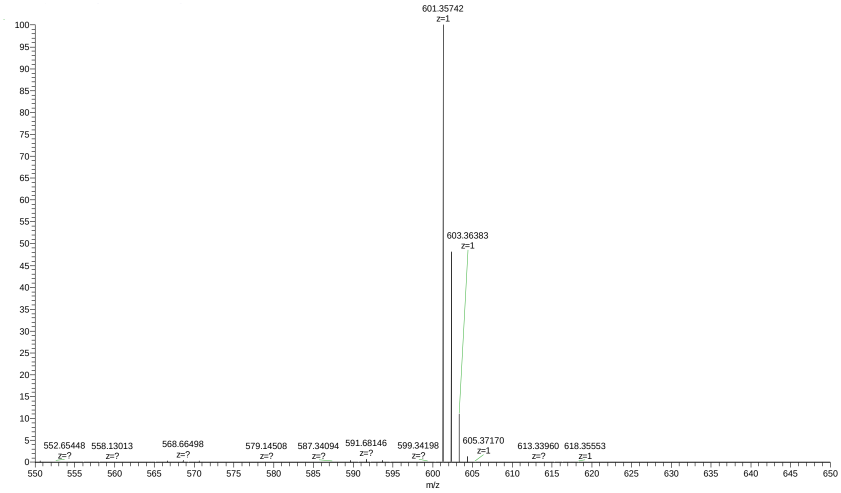

Figure S40. HR-ESI-MS spectrum of compound **1,6-DNPy-L**.

Computational data for compounds 1,8-DNPy-B, 1,6-DNPy-B, 1,8-DNPy-L, 1,6-DNPy-L:

Compound 1,8-DNPy-B

E = - 5362.2129846984 Hartrees

Center Number	Atomic Number	Atomic Type	Coordinates (Angstroms)		
			X	Y	Z
1	6	0	-0.715053	-2.013528	-0.032653
2	6	0	0.714765	-2.013595	0.032531
3	6	0	1.454991	-0.798394	-0.006588
4	6	0	0.68516	0.402692	-0.001957
5	6	0	-0.685229	0.402754	0.001964
6	6	0	-1.455167	-0.798261	0.006573
7	6	0	-1.41224	-3.263252	-0.155296
8	6	0	-0.672019	-4.497609	-0.088565
9	6	0	0.671507	-4.497678	0.088173
10	6	0	1.411836	-3.263394	0.155053
11	6	0	2.778545	-3.25387	0.375444
12	6	0	3.537465	-2.065147	0.347509
13	6	0	2.900309	-0.834916	0.005917
14	6	0	-2.900489	-0.834649	-0.005898
15	6	0	-3.537765	-2.064795	-0.347576
16	6	0	-2.778953	-3.253583	-0.375659
17	6	0	-7.026659	-0.759279	-0.815663
18	6	0	-5.656684	-0.843739	-0.475203
19	6	0	-4.956722	-2.065435	-0.647896
20	6	0	-5.668142	-3.18259	-1.143543
21	6	0	-7.007792	-3.082265	-1.472691

22	6	0	-7.693358	-1.861256	-1.312134
23	6	0	7.00737	-3.082998	1.472676
24	6	0	7.693038	-1.862028	1.312259
25	6	0	7.026448	-0.759956	0.815856
26	6	0	5.656482	-0.844277	0.475329
27	6	0	5.667726	-3.183187	1.14346
28	6	0	4.956413	-2.065933	0.647881
29	7	0	5.065206	0.256201	-0.088215
30	6	0	3.781115	0.27526	-0.347735
31	6	0	-3.781182	0.275582	0.347854
32	7	0	-5.06529	0.256647	0.088397
33	6	0	3.351909	1.496783	-1.171818
34	6	0	3.403589	2.771576	-0.291284
35	6	0	2.77998	4.026917	-0.917231
36	6	0	2.805788	5.238796	0.024654
37	6	0	2.192709	6.498549	-0.595484
38	6	0	4.241963	1.623759	-2.431599
39	6	0	4.106563	0.447209	-3.404385
40	6	0	-3.351845	1.49701	1.172006
41	6	0	-3.403403	2.771843	0.291518
42	6	0	-2.779321	4.027026	0.917306
43	6	0	-2.80479	5.238817	-0.0247
44	6	0	-2.191137	6.498397	0.595223
45	6	0	-4.241878	1.623973	2.431807
46	6	0	-4.106711	0.447235	3.4044
47	1	0	1.18307	1.359649	0.043944
48	1	0	-1.183058	1.359755	-0.043944
49	1	0	-1.222813	-5.431486	-0.167512
50	1	0	1.222219	-5.431613	0.166993
51	1	0	3.276403	-4.203122	0.542572
52	1	0	-3.276902	-4.202769	-0.542892
53	1	0	-7.52509	0.193016	-0.663477
54	1	0	-5.159751	-4.128133	-1.301268
55	1	0	-7.532107	-3.950004	-1.86346
56	1	0	-8.745541	-1.792179	-1.574448
57	1	0	7.531601	-3.950813	1.863389
58	1	0	8.745215	-1.793057	1.574626
59	1	0	7.524963	0.192312	0.663775
60	1	0	5.159257	-4.128705	1.301079
61	1	0	2.329704	1.35149	-1.530961
62	1	0	2.896716	2.570477	0.664196
63	1	0	4.453081	2.959377	-0.030939
64	1	0	3.304336	4.289351	-1.84534
65	1	0	1.738591	3.816286	-1.206389

66	1	0	2.271608	4.989704	0.952748
67	1	0	3.844654	5.443899	0.319058
68	1	0	2.232236	7.346048	0.098346
69	1	0	1.141313	6.337751	-0.864868
70	1	0	2.726058	6.790597	-1.508545
71	1	0	3.968959	2.547242	-2.956581
72	1	0	5.284809	1.727743	-2.114145
73	1	0	4.722495	0.607107	-4.296741
74	1	0	3.068304	0.320045	-3.736171
75	1	0	4.428008	-0.494651	-2.946321
76	1	0	-2.329649	1.351583	1.531122
77	1	0	-2.896768	2.570614	-0.664062
78	1	0	-4.452898	2.959911	0.031379
79	1	0	-3.303502	4.289734	1.845436
80	1	0	-1.737978	3.816061	1.206384
81	1	0	-2.270833	4.989424	-0.952841
82	1	0	-3.843617	5.444289	-0.318982
83	1	0	-2.230439	7.345839	-0.098689
84	1	0	-1.139764	6.337225	0.86447
85	1	0	-2.724242	6.790742	1.508331
86	1	0	-3.968702	2.547316	2.956942
87	1	0	-5.284705	1.728206	2.114366
88	1	0	-4.722637	0.607094	4.296767
89	1	0	-3.068483	0.319828	3.736191
90	1	0	-4.428312	-0.494492	2.94617

Compound 1,6-DNPy-B

E = - 5254.6658545223 Hartrees

Center Number	Atomic Number	Atomic Type	Coordinates (Angstroms)		
			X	Y	Z
1	6	0	-0.587371	-0.410958	-0.383261
2	6	0	0.58733	0.410969	-0.383257
3	6	0	0.444965	1.830905	-0.473018
4	6	0	-0.874036	2.380196	-0.5505
5	6	0	-1.981531	1.592202	-0.469684
6	6	0	-1.892056	0.162632	-0.3226
7	6	0	-0.44501	-1.830893	-0.47304
8	6	0	0.873992	-2.380183	-0.550524
9	6	0	1.981488	-1.59219	-0.4697
10	6	0	1.892016	-0.16262	-0.322606
11	6	0	3.040311	0.693976	-0.215763
12	6	0	2.874378	2.094296	-0.472094
13	6	0	1.581192	2.627618	-0.573689

14	6	0	-3.040351	-0.693964	-0.215763
15	6	0	-2.874423	-2.09428	-0.472121
16	6	0	-1.581237	-2.627604	-0.573725
17	6	0	-6.506298	-3.039775	-0.649335
18	6	0	-5.317143	-2.307313	-0.435008
19	6	0	-4.056966	-2.923576	-0.638879
20	6	0	-4.030524	-4.280296	-1.032414
21	6	0	-5.204355	-4.984089	-1.239201
22	6	0	-6.452728	-4.359938	-1.052062
23	6	0	6.506248	3.039806	-0.64932
24	6	0	6.452671	4.359981	-1.052008
25	6	0	5.204295	4.984135	-1.239112
26	6	0	4.030467	4.280334	-1.032332
27	6	0	5.317096	2.307337	-0.434999
28	6	0	4.056916	2.923602	-0.638839
29	6	0	-4.380098	-0.259639	0.183186
30	7	0	-5.431335	-1.024999	0.043543
31	6	0	-4.679401	1.050359	0.924813
32	6	0	-5.521254	1.987721	0.021345
33	6	0	-5.672015	3.426712	0.534311
34	6	0	-6.45399	4.326619	-0.432609
35	6	0	-6.611393	5.763773	0.074542
36	6	0	-5.39038	0.749624	2.265745
37	6	0	-4.532916	-0.04393	3.257592
38	7	0	5.431297	1.025011	0.04352
39	6	0	4.380064	0.259646	0.183166
40	6	0	4.679378	-1.050385	0.924735
41	6	0	5.521231	-1.987709	0.021229
42	6	0	5.672121	-3.42667	0.534242
43	6	0	6.454195	-4.326535	-0.432636
44	6	0	6.611747	-5.763651	0.074575
45	6	0	5.390373	-0.749746	2.265677
46	6	0	4.532861	0.04358	3.257666
47	1	0	-0.979802	3.452026	-0.698579
48	1	0	-2.95091	2.052384	-0.595609
49	1	0	0.97976	-3.452011	-0.698606
50	1	0	2.950862	-2.052376	-0.595627
51	1	0	1.44493	3.693705	-0.721098
52	1	0	-1.444977	-3.693689	-0.72115
53	1	0	-7.449175	-2.529961	-0.477466
54	1	0	-3.08357	-4.781534	-1.203945
55	1	0	-5.159867	-6.023439	-1.55283
56	1	0	-7.369305	-4.919398	-1.21798
57	1	0	7.449127	2.529989	-0.477475

58	1	0	7.369246	4.919447	-1.217921
59	1	0	5.159801	6.023495	-1.552707
60	1	0	3.083511	4.781579	-1.203833
61	1	0	-3.739024	1.548279	1.176565
62	1	0	-5.073352	2.022215	-0.982905
63	1	0	-6.50809	1.527909	-0.116846
64	1	0	-6.176749	3.431756	1.509309
65	1	0	-4.675384	3.861898	0.706599
66	1	0	-5.94903	4.335887	-1.409197
67	1	0	-7.446733	3.889329	-0.609381
68	1	0	-7.170428	6.380912	-0.638073
69	1	0	-5.634746	6.238296	0.231767
70	1	0	-7.148333	5.790181	1.030785
71	1	0	-5.674065	1.702817	2.728399
72	1	0	-6.316285	0.204557	2.053645
73	1	0	-5.067429	-0.191616	4.202968
74	1	0	-3.595415	0.47923	3.48467
75	1	0	-4.275582	-1.034445	2.866381
76	1	0	3.739007	-1.548324	1.176474
77	1	0	5.073284	-2.022258	-0.982999
78	1	0	6.508031	-1.527837	-0.117019
79	1	0	6.17684	-3.431629	1.50925
80	1	0	4.675528	-3.861943	0.706533
81	1	0	5.949239	-4.335896	-1.409226
82	1	0	7.446894	-3.88915	-0.609423
83	1	0	7.170848	-6.38076	-0.638015
84	1	0	5.635149	-6.238271	0.231816
85	1	0	7.148688	-5.789965	1.030819
86	1	0	5.674181	-1.702973	2.728188
87	1	0	6.316211	-0.204551	2.053614
88	1	0	5.067393	0.191194	4.203043
89	1	0	3.595424	-0.479711	3.484708
90	1	0	4.275408	1.03412	2.8666

Compound 1,8-DNPy-L
E = - 4961.6936826126 Hartrees

Center Number	Atomic Number	Atomic Type	Coordinates (Angstroms)		
			X	Y	Z
1	6	0	-0.693058	-2.501102	0.180881
2	6	0	0.693405	-2.501028	-0.181057
3	6	0	1.427336	-1.285796	-0.313231
4	6	0	0.669159	-0.089467	-0.146385
5	6	0	-0.669157	-0.089544	0.145847

6	6	0	-1.427157	-1.285954	0.312918
7	6	0	-1.352148	-3.753021	0.426538
8	6	0	-0.641811	-4.988236	0.214303
9	6	0	0.64249	-4.988171	-0.21424
10	6	0	1.352674	-3.752883	-0.426564
11	6	0	2.654884	-3.751201	-0.893001
12	6	0	3.404965	-2.566571	-1.038297
13	6	0	2.837723	-1.319625	-0.638323
14	6	0	-2.837518	-1.319936	0.638106
15	6	0	-3.404552	-2.56691	1.038281
16	6	0	-2.654332	-3.751461	0.893047
17	6	0	-6.778497	-1.334726	2.132206
18	6	0	-5.477211	-1.383306	1.58112
19	6	0	-4.75127	-2.599783	1.577541
20	6	0	-5.366984	-3.748851	2.126218
21	6	0	-6.639545	-3.684177	2.664385
22	6	0	-7.352561	-2.468666	2.670653
23	6	0	6.640288	-3.683589	-2.66391
24	6	0	7.353104	-2.46796	-2.670325
25	6	0	6.778815	-1.334027	-2.132106
26	6	0	5.477491	-1.382723	-1.581117
27	6	0	5.367692	-3.748379	-2.12584
28	6	0	4.751741	-2.599318	-1.577416
29	7	0	4.982419	-0.237747	-1.012418
30	6	0	3.758901	-0.18417	-0.551204
31	6	0	-3.758887	-0.184626	0.550907
32	7	0	-4.982358	-0.238333	1.012219
33	6	0	3.428936	1.116261	0.176806
34	6	0	4.654909	1.890802	0.680938
35	6	0	4.259802	3.157905	1.450859
36	6	0	5.466797	3.953957	1.963951
37	6	0	5.082253	5.222678	2.735668
38	6	0	6.288817	6.017673	3.250945
39	6	0	5.896151	7.283238	4.019959
40	6	0	-3.429167	1.115775	-0.177257
41	6	0	-4.655275	1.890177	-0.681236
42	6	0	-4.260384	3.157348	-1.451158
43	6	0	-5.467494	3.953397	-1.963955
44	6	0	-5.083122	5.222222	-2.735594
45	6	0	-6.289794	6.017293	-3.250483
46	6	0	-5.897295	7.282953	-4.019427
47	1	0	1.139189	0.87035	-0.282637
48	1	0	-1.139331	0.870226	0.281914
49	1	0	-1.169224	-5.921351	0.395344

50	1	0	1.170022	-5.921234	-0.395202
51	1	0	3.11347	-4.706428	-1.124141
52	1	0	-3.11279	-4.706722	1.124297
53	1	0	-7.299089	-0.382481	2.108643
54	1	0	-4.8354	-4.694182	2.155539
55	1	0	-7.089076	-4.577417	3.08947
56	1	0	-8.351625	-2.428241	3.095887
57	1	0	7.090006	-4.57683	-3.088794
58	1	0	8.352194	-2.42744	-3.095489
59	1	0	7.299256	-0.381696	-2.10865
60	1	0	4.836275	-4.693808	-2.155024
61	1	0	2.857437	1.778401	-0.491149
62	1	0	2.769972	0.89947	1.025615
63	1	0	5.25681	1.236978	1.325515
64	1	0	5.298016	2.149013	-0.166116
65	1	0	3.648865	3.805877	0.803842
66	1	0	3.616461	2.888684	2.302313
67	1	0	6.078797	3.307279	2.61009
68	1	0	6.109023	4.226017	1.113229
69	1	0	4.471195	5.870764	2.089439
70	1	0	4.438709	4.950677	3.585952
71	1	0	6.898744	5.370656	3.897516
72	1	0	6.932047	6.29018	2.401956
73	1	0	6.779194	7.827526	4.3741
74	1	0	5.281252	7.040821	4.895636
75	1	0	5.315104	7.966598	3.388318
76	1	0	-2.857642	1.778048	0.490546
77	1	0	-2.77027	0.898967	-1.026127
78	1	0	-5.257192	1.236315	-1.325767
79	1	0	-5.298308	2.148288	0.165903
80	1	0	-3.649346	3.805299	-0.804209
81	1	0	-3.617189	2.888207	-2.30275
82	1	0	-6.079591	3.30678	-2.610064
83	1	0	-6.109585	4.225344	-1.113093
84	1	0	-4.471908	5.870213	-2.089414
85	1	0	-4.43977	4.950329	-3.58606
86	1	0	-6.899888	5.370372	-3.896992
87	1	0	-6.932818	6.289698	-2.401304
88	1	0	-6.780416	7.827305	-4.373277
89	1	0	-5.282616	7.040643	-4.89529
90	1	0	-5.316079	7.966217	-3.387836

Compound 1,6-DNPy-L

E = - 4909.8685609735 Hartrees

Center Number	Atomic Number	Atomic Type	Coordinates (Angstroms)		
			X	Y	Z
1	6	0	0.52689	-0.486765	-1.172788
2	6	0	-0.526975	0.48681	-1.172703
3	6	0	-0.19058	1.873751	-1.245793
4	6	0	1.190207	2.238989	-1.314866
5	6	0	2.180448	1.307634	-1.248273
6	6	0	1.901214	-0.09915	-1.120922
7	6	0	0.190502	-1.873685	-1.246152
8	6	0	-1.190281	-2.238923	-1.315239
9	6	0	-2.180528	-1.30759	-1.248421
10	6	0	-1.901293	0.099176	-1.120926
11	6	0	-2.924307	1.10483	-1.022203
12	6	0	-2.558615	2.47517	-1.229854
13	6	0	-1.203845	2.822959	-1.321871
14	6	0	2.924202	-1.104818	-1.022215
15	6	0	2.558533	-2.475134	-1.230057
16	6	0	1.203771	-2.822885	-1.322348
17	6	0	6.006516	-3.965188	-1.303885
18	6	0	4.93941	-3.051664	-1.153651
19	6	0	3.603994	-3.478915	-1.35076
20	6	0	3.37737	-4.833601	-1.682609
21	6	0	4.433263	-5.716673	-1.829029
22	6	0	5.759162	-5.28136	-1.642295
23	6	0	-6.006592	3.965236	-1.303862
24	6	0	-5.759194	5.281421	-1.642186
25	6	0	-4.433279	5.71671	-1.828896
26	6	0	-3.377415	4.833608	-1.682477
27	6	0	-4.939514	3.051667	-1.153643
28	6	0	-3.604085	3.478925	-1.350641
29	6	0	4.325943	-0.850309	-0.670783
30	7	0	5.246817	-1.773123	-0.75581
31	7	0	-5.246965	1.773089	-0.755973
32	6	0	-4.326078	0.850283	-0.670964
33	6	0	4.81477	0.462547	-0.062887
34	6	0	6.047813	0.317211	0.840785
35	6	0	6.476444	1.65642	1.454609
36	6	0	7.712233	1.540152	2.356279
37	6	0	8.151434	2.875085	2.971262
38	6	0	9.388328	2.759845	3.871237
39	6	0	9.82164	4.097645	4.47944
40	6	0	-4.814868	-0.462558	-0.063052
41	6	0	-6.048048	-0.317263	0.840444

42	6	0	-6.476378	-1.656414	1.454589
43	6	0	-7.712265	-1.540234	2.356144
44	6	0	-8.151114	-2.875115	2.971489
45	6	0	-9.388042	-2.759968	3.87143
46	6	0	-9.821004	-4.097736	4.479955
47	1	0	1.441035	3.289273	-1.441121
48	1	0	3.200674	1.640569	-1.355381
49	1	0	-1.441108	-3.289195	-1.441603
50	1	0	-3.200763	-1.640521	-1.355403
51	1	0	-0.918746	3.862707	-1.439959
52	1	0	0.918656	-3.862584	-1.440802
53	1	0	7.013075	-3.594164	-1.137857
54	1	0	2.368336	-5.195148	-1.850885
55	1	0	4.234991	-6.751565	-2.094236
56	1	0	6.582083	-5.981043	-1.759809
57	1	0	-7.013168	3.594227	-1.13791
58	1	0	-6.582094	5.981135	-1.759664
59	1	0	-4.234984	6.751598	-2.094106
60	1	0	-2.368363	5.195078	-1.850807
61	1	0	5.062836	1.171784	-0.867188
62	1	0	4.003062	0.92514	0.50965
63	1	0	5.827925	-0.403865	1.63928
64	1	0	6.87166	-0.11553	0.265087
65	1	0	6.683804	2.378887	0.650313
66	1	0	5.643766	2.081564	2.035215
67	1	0	7.506715	0.819369	3.161605
68	1	0	8.544733	1.115795	1.775862
69	1	0	8.356273	3.596769	2.166041
70	1	0	7.319164	3.299358	3.552864
71	1	0	9.183687	2.040814	4.677385
72	1	0	10.219857	2.335478	3.290686
73	1	0	10.705084	3.980445	5.117566
74	1	0	9.022702	4.531548	5.093514
75	1	0	10.069797	4.826944	3.698281
76	1	0	-5.062671	-1.171907	-0.867338
77	1	0	-4.00324	-0.925038	0.509689
78	1	0	-5.828441	0.404091	1.638762
79	1	0	-6.871942	0.115125	0.264543
80	1	0	-6.683485	-2.37918	0.650497
81	1	0	-5.643624	-2.081156	2.035381
82	1	0	-7.507007	-0.819162	3.161281
83	1	0	-8.544839	-1.116273	1.775545
84	1	0	-8.355748	-3.597086	2.166471
85	1	0	-7.318733	-3.298988	3.553221

86	1	0	-9.183584	-2.040694	4.677409
87	1	0	-10.219675	-2.335948	3.290776
88	1	0	-10.704464	-3.980611	5.118073
89	1	0	-9.021941	-4.531288	5.094113
90	1	0	-10.068991	-4.827278	3.698969

References

1. C. A. Tolman, W. C. Seidel and D. H. Gerlach, *J. Am. Chem. Soc.*, 1971, **94**, 2669–2676.
2. A. Stanger, *J. Org. Chem.* 2006, **71**, 883–893.
3. A. Stanger, *J. Org. Chem*. 2010, **75**, 2281–2288.
4. R. Gershoni-Poranne, A. Stanger, *Chem. Eur. J.* 2014, **20**, 5673–5688.
5. D. Geuenich, K. Hess, F. Köhler, R. Herges, *Chem. Rev.* 2005, **105**, 3758–3772.
6. A. G. Crawford, Z. Liu, I. A. I. Mkhalid, M. Thibault, N. Schwarz, G. Alcaraz, A. Steffen, J. C. Collings, A. S. Batsanov, J. A. K. Howard, and T. B. Marder, *Chem. Eur. J.*, 2012, **18**, 5022–5035.
